# Comprehensibility of Contraindications in German, UK and US Summaries of Product Characteristics/Prescribing Information—A Comparative Qualitative and Quantitative Analysis

**DOI:** 10.3390/jcm11144167

**Published:** 2022-07-18

**Authors:** Melanie I. Then, Wahram Andrikyan, Martin F. Fromm, Renke Maas

**Affiliations:** Institute of Experimental and Clinical Pharmacology and Toxicology, Friedrich-Alexander-Universität Erlangen-Nürnberg, Fahrstr. 17, 91054 Erlangen, Germany; wahram.andrikyan@fau.de (W.A.); martin.fromm@fau.de (M.F.F.); renke.maas@fau.de (R.M.)

**Keywords:** medication safety, contraindications, Summary of Product Characteristics, Prescribing Information

## Abstract

Contraindications (CIs) in Summaries of Product Characteristics (SmPCs)/Prescribing Information (PI) that lack clarity may pose a risk to medication safety and increase the risk for adverse drug reactions. We assessed and compared SmPCs/PI from three major drug markets regarding comprehensibility from the prescriber perspective, as well as usability in clinical decision support systems. 158 drugs met the following inclusion criteria: marketed in Germany (DE), United Kingdom (UK) and United States (US) and belonged to the 100 most recently FDA approved and/or 100 most frequently prescribed drugs in either country. In the 474 (3 × 158) SmPCs/PI all expressions for absolute CIs were identified, divided into 3999 stand-alone terms and evaluated according to ‘clarity’ and ‘codability’. The average number of absolute CIs per drug differed drastically between the three markets (DE: 11.7, UK: 9.0, US: 4.6). Expressions were frequently unclear (DE: 27.2% (95% CI 25.2–29.2%), UK: 28.5% (26.2–30.9%), US: 22.6% (19.7–25.8%)). Moreover, 60.9% (58.6–63.1%), 63.6% (61.0–66.0%), and 64.7% (61.2–68.1%) of the expressions were not codable in DE, UK, and US, respectively. Taken together, in three major drug markets, statements regarding CIs in SmPCs/PI substantially differ in frequency and frequently lack clarity and codability which poses an unnecessary obstacle to medication safety.

## 1. Introduction

Summaries of Product Characteristics (SmPCs) and Prescribing Information (PI) are one of the main sources of information that prescribers rely on for safe and effective drug prescribing [1,2]. Besides other knowledge bases, the content of SmPCs/PI is essential for the development of electronic clinical decision support systems (CDSS) [3,4]. According to EMA (European Medicines Agency) and FDA (US Food and Drug Administration) guidelines, in the contraindications (CIs), all situations should be mentioned when the drug must not be given due to safety reasons including diagnoses, demographic factors, predispositions or use of other drugs [5,6].

Unclear expressions within SmPCs/PI that leave too much room for interpretation pose a risk to medication safety, especially when the sections about CIs, adverse events or dosing are affected [7,8]. Earlier studies regarding lack of clarity of medication information often focused on medical websites and patient information leaflets with the conclusion that comprehensibility was especially low for CIs, interactions and precautions [9,10].

For SmPCs/PI there is only limited data available with respect to the extent and type of CI statements that are unclear to prescribers. In addition to unclear expressions in medical information, significant discrepancies have also been observed when comparing the SmPCs/PI for different countries. Comparative studies in the past have analyzed information gaps in one country compared to another, quantitative measurements of words, or evaluated, for example, discrepancies in approved maximal dose [11,12]. More specific studies focused on dose adjustment in patients with renal impairment or inconsistencies and ambiguities in contraindications regarding liver diseases [8,13,14].

Mapping, but also the digital transfer, of information is becoming increasingly important in our digital age. Previous studies on SmPCs/PI have focused on the transfer of free text into semantic labels or annotations using for example machine learning tools or by comparing different NLP (natural language processing) techniques [15,16]. There is hardly any data, especially for the CIs section, on the feasibility of the transfer from the expression as provided in the SmPC/PI to a universal code with a matching definition, which would make an international standardization of shared coding of regionally used expressions possible.

Furthermore, different legal systems and regulatory requirements might also have an influence on the content of the SmPCs/PI leading to heterogenous treatment of patients in different countries. For example, the definition of a CI itself varies between countries, and the topic “hypersensitivity” has a different significance when comparing US with EU requirements [6,17].

The focus of our study was to analyze all absolute CIs for a representative sample of drugs regarding clinical clarity and codability from a qualitative as well as quantitative point of view. Therefore, we created an evaluation system to assess the comprehensibility and clinical applicability from the prescriber perspective, as well as usability, in CDSS and compared the results for the three major drug markets, Germany, UK and US.

## 2. Materials and Methods

### 2.1. Drug Selection

**Figure 1:**
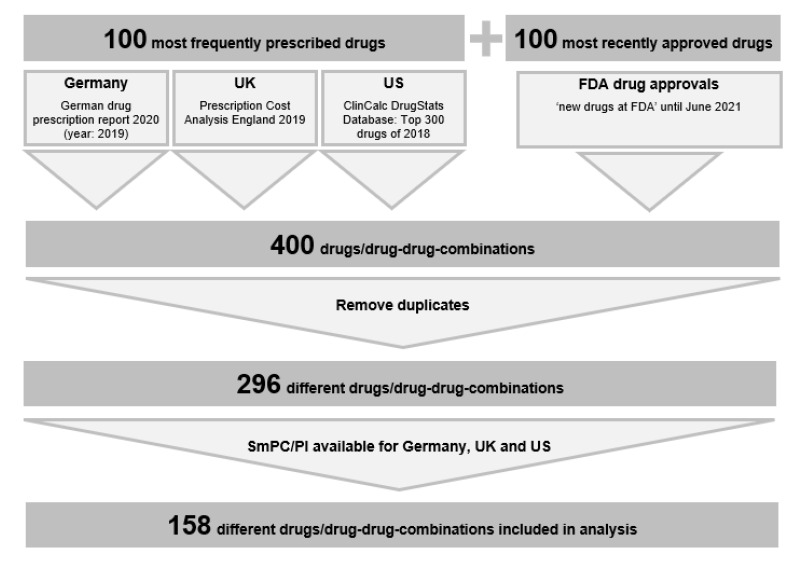
Selection of drugs for analysis.

To obtain a representative sample, the 100 most frequently prescribed drugs and drug–drug combinations in Germany, England and US in addition to the 100 latest novel drug approvals by the FDA until June 2021 formed the basis for the drug selection [18]. The top 100 drugs from Germany were taken from the 3000 most frequently prescribed medicines in the outpatient sector based on prescription numbers for the year 2019, available via the ‘PharmMaAnalyst’ database for the German Drug Prescription Report [19,20]. The top 100 drugs from England were taken from the most frequently prescribed chemical substances by dispensed items in the Prescription Cost Analysis 2019, which covers all prescriptions dispensed in the community in England [21]. The top 100 drugs from the US were taken from the top 300 drugs of 2018 in the ClinCalc DrugStats Database, which covers all US prescription data estimates by the Medical Expenditure Panel Survey [22]. Of these 400 drugs and drug–drug combinations, 104 were excluded because of duplication and 138 because there was no SmPC/PI available for all three markets (Germany, UK, and US), resulting in 158 drugs included in the analysis (Figure 1).

### 2.2. SmPCs/PI

For each drug a SmPC/PI was identified in each country (i.e., 3 × 158 SmPCs/PI). The SmPCs for Germany were extracted from the German databases ‘Fachinfo’ or ‘Gelbe Liste Pharmindex’ [23,24]. The SmPCs for UK were taken from the ‘electronic medicines compendium’ database [25]. The PI for US were extracted from the FDA Professional Drug Information available via the ‘Drugs.com’ database and the ‘Drugs@FDA’ website [26,27]. For each drug, one exemplary SmPC/PI was selected, preferably by the original manufacturer or, if not available, the latest by date. For the complete list of all 474 included SmPCs/PI, see Table S1 (DE), Table S2 (UK) and Table S3 (US).

### 2.3. Extraction of Absolute CIs

The German CIs section was translated into English for the evaluations within the frame of this analysis. From the text in the CIs section (DE/UK: SmPC chapter ‘4.3 Contraindications’, US: PI chapter ‘Contraindications’) all distinct absolute CIs were identified. Terms containing wording such as ‘should not’ or ‘is not recommended’ were not considered as absolute CIs. Aggregated expressions covering multiple clinical conditions were divided into separate individual stand-alone statements (e.g., ‘recent brain or spinal injury’ was divided into ‘recent brain injury’ and ‘recent spinal injury’). If a general term was specified with an example, both the general term as well as the example were counted as individual CIs. For an exemplary extraction of all individual absolute CIs form the CIs section, see Table S4 for UK SmPC of rivaroxaban.

### 2.4. Categorization of the Individual CI Statements

Based on the content, the individual CI statements were assigned to one or more of the three broad categories: patient-related, medication-related and disease-related. CIs related to pregnancy/birth/lactation, age and female sex were categorized as patient-related. The fourth topic ‘other’ within the patient-related CIs groups contains rather rare terms such as ‘Asian ancestry’, ‘in siblings’ or ‘smoking’. Statements referring to drug, drug-family, administration and dose were considered as drug-related. CIs referring to musculoskeletal system, genetic disorders, eyes/nose/throat/ears, urogenital system, infections, skin, psyche, neoplasia, kidney, nervous system, respiratory system, metabolism, gastrointestinal tract, laboratory discrepancies, liver, blood/blood-formation, cardiovascular system and immune system were considered as disease-related. One CI could be assigned to more than one of the three broad categories and its subcategories.

### 2.5. Analysis of CI Statements

The comprehensibility and usability of each absolute CI statement was classified by using the two criteria ‘clarity’ (prescriber perspective) and ‘codability’ (machine perspective).

### 2.6. Clarity

An expression was defined as clear if it could safely be assumed that all prescribers, or at least an overwhelming majority, came to the same conclusion as to whether it was present or not when applying it to the same patient. In all other cases, the expression was considered unclear. The following factors were considered indicative of unclear expressions:

1. The term covers a range of diagnoses of which only some can reasonably be assumed to pose a clinically relevant risk when present in a patient taking the drug (‘liver disease’, ‘vascular aneurism’).
2. No clear cut off values or definitions are provided (‘impaired renal function’, ‘conditions with increased potassium losses’), or definitions are clear but cover a (too) broad range of conditions of which only some are specific or relevant only for the intended CI (‘vascular disease’).
3. The term requires complex clinical judgements, with different prescribers likely coming to different conclusions for the same case because no objective commonly agreed criteria are provided (‘condition if considered a significant risk factor for major bleeding’).

Further examples are given in the results section.

### 2.7. Codability

The codability of a term (e.g., how well it was deemed to be translatable into a commonly used unique code) was classified by a three-level approach (simple, complex, not codable). A term fulfilled the simple codability level if it could be translated directly into one defined code or a combination of codes (‘contraindicated in combination with simvastatin’, ‘contraindicated in combination with ACE-inhibitors’). Simple codes within this work included the common medical code systems ATC (Anatomical Therapeutic Chemical Classification System), ICD-10, PZN (Germany: Pharma Central Number, analogously for UK and US), OPS (Germany: Operation and Procedure Classification System, analogously for UK and US), as well as the simple digitally recordable demographic parameters (age, sex) or pharmacogenetic factors. For the disease-related CIs we checked in detail, if there was a specific ICD-10 code available for the expression used in the SmPC/PI [28,29].

We assigned terms to the complex codability level if the mapping in a digital system would only be possible if an expert/intelligence created an algorithm. Such an algorithm can be described for example by simple codes plus an expert-defined cut-off (e.g., for term ‘do not use in children before onset of puberty’), using a reference database with the status of science (‘CYP3A4 inhibitors’), or by using a combination of simple codes with more complex digitally recordable data such as ECG (electrocardiogram) data or LOINC (Logical Observation Identifiers Names and Codes) parameters. All other terms, which could currently not be defined by the simple or complex levels were classified as not codable (e.g., ‘abdominal pain of unknown origin’, ‘clinically significant bleeding’).

### 2.8. Statistical Analysis

Key parameters are presented with 95% confidence intervals (CI) calculated for a sample proportion using Epitools epidemiological calculators with ‘Wilson’ score interval [30]. A random sample of 10% of all CI items was evaluated by a second analyst to assess the inter-rater reliability for the two criteria ‘clarity’ and ‘codability’ with ‘Cohen’s kappa coefficient of agreement’ (ĸ) using ‘idostatistics’ free calculator [31].

## 3. Results

### 3.1. SmPCs/PI Not Listing any CI

**Figure 2:**
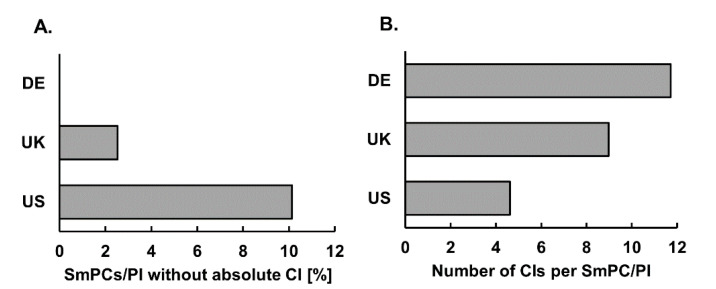
Quantitative analysis of CIs in DE, UK and US SmPCs/PI. (**A**). Percentage of SmPCs/PI among 158 analyzed SmPCs/PI without any absolute CI in Germany, UK and US. (**B**). Average number of absolute CIs per SmPC/PI in German, UK and US PI among 158 analyzed SmPCs/PI. CI: contraindication, DE: Germany, PI: Prescribing Information, SmPC: Summary of Product Characteristics.

For the 158 drugs with SmPC/PI available in all three countries, all German SmPCs listed at least one absolute CI, a total of four (2.5%, 95% CI 1.0–6.3%) SmPCs from the UK (allopurinol, atenolol, aspirin, clonidine) contained no absolute CI, whilst 16 (10.1%, 95% CI 6.3–15.8%) US PI had no absolute CI (belantamab mafodotin, bempedoic acid, clonidine, dostarlimab, elexacaftor/ivacaftor/tezacaftor, estradiol, ethinylestradiol/norethisterone, hydrocortisone, levonogestrel/ethinylestradiol, olanzapine, osilodrostat, pemigatinib, risdiplam, selpercatinib, tivozanib, topiramate) (Figure 2A).

### 3.2. Number of Individual Absolute CIs

From all 474 analyzed SmPCs/PI (158 per country), we extracted in total 3999 individual terms/expressions of absolute CIs (on average 11.7 (SD = 12.2) per SmPC in Germany, 9.0 (SD = 8.5) per SmPC in the UK and 4.6 (SD = 4.9) per PI in the US (Figure 2B)).

### 3.3. Categories of CI

**Figure 3:**
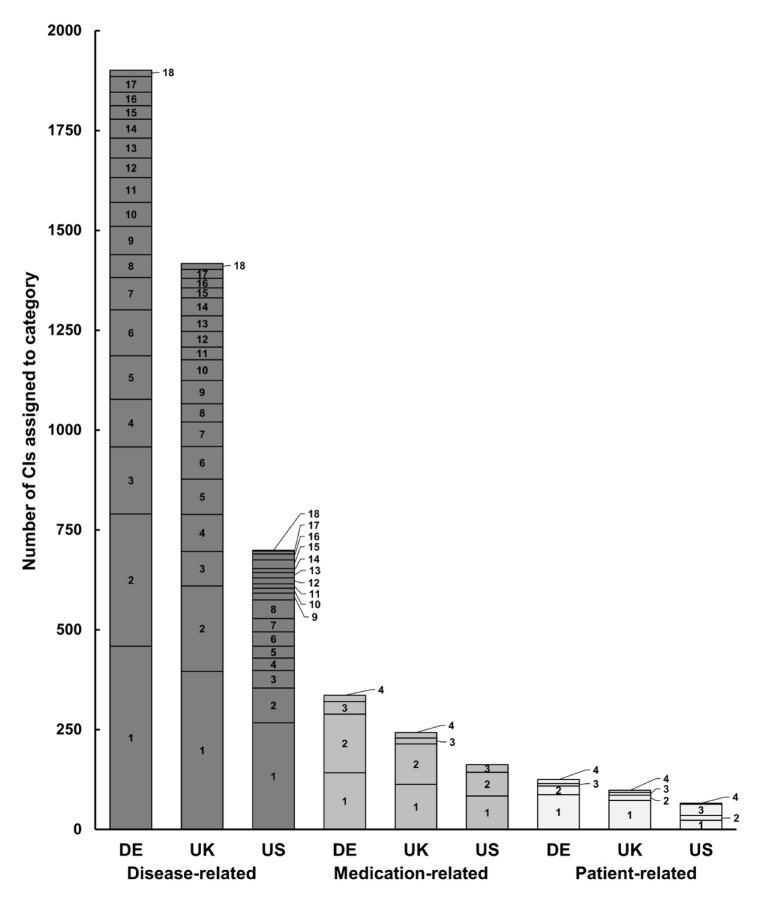
Number of individual absolute CIs assigned to each category, separately shown for German and UK SmPCs and US PI. Dark grey: disease-related CIs: 1: immune system, 2: cardiovascular system, 3: blood/blood formation, 4: liver, 5: gastrointestinal tract, 6: laboratory discrepancies, 7: metabolism, 8: respiratory system, 9: nervous system, 10: kidney, 11: neoplasia, 12: skin, 13: psyche, 14: infections, 15: urogenital system, 16: eyes/nose/throat/ears, 17: genetic disorders, 18: musculoskeletal system; medium grey: medication-related CIs: 1: drug, 2: drug-family, 3: administration, 4: dose; light grey: patient-related CIs: 1: pregnancy/birth/lactation, 2: age, 3: female sex, 4: other. CI: contraindication, DE: Germany, PI: Prescribing Information, SmPC: Summary of Product Characteristics.

Most of the CIs (DE + UK + US) were related to diseases (3341; 83.5%), followed by medication (704; 17.6%) and patient (270; 6.8%) (Figure 3). Overall, most frequent disease-related CIs were related to immune system (1122; 33.6%), cardiovascular system (632; 18.9%) and blood/blood formation (298; 8.9%), whilst CIs were least frequently related to musculoskeletal system (33; 1.0%), genetic disorders (68; 2.0%); and eyes/nose/throat/ears (73; 2.2%) (Figure 3). While 16 German and 14 UK SmPCs listed dose-related CIs, none were listed in the analyzed US PI (Figure S1A). Whilst in the German and UK SmPCs pregnancy/birth/lactation was the most frequent topic among the patient-related CIs, in US PI it was female sex (Figure S1B).

On average across all three markets each individual CI statement was assigned to 1.26 subcategories, as many CIs contained combined terms such as ‘contraindicated in women over 35 years with migraine’ or ‘contraindicated in patients with diabetes mellitus with vascular changes’.

### 3.4. Clarity

**Figure 4:**
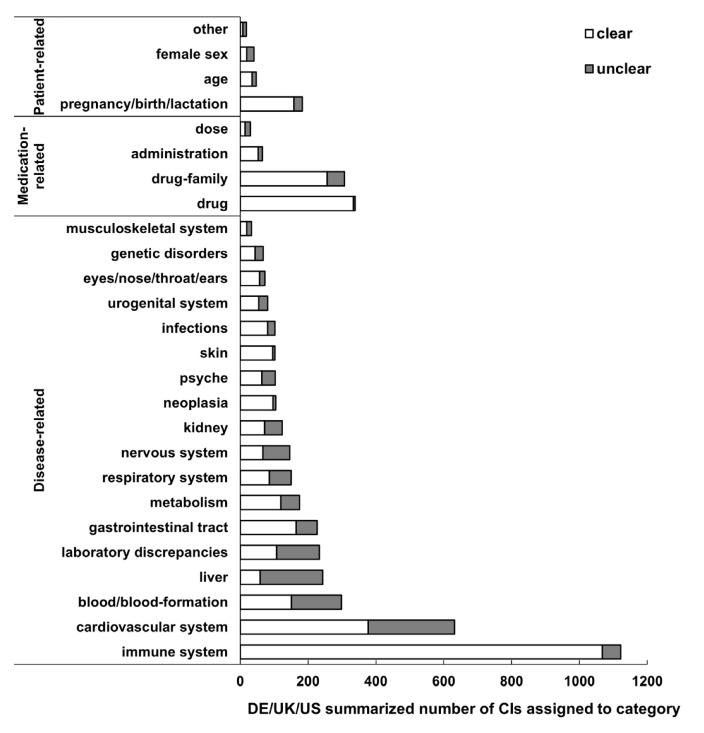
The average clarity from the prescriber perspective of all absolute CIs in German and UK SmPCs and US PI is shown in total numbers of individual CIs assigned to each category, summarized for Germany, UK and US. The three categories patient-, medication- and disease-related CIs are sorted by frequency among all CIs, as well as the individual subcategories. White: clear, grey: unclear. CI: contraindication, DE: Germany.

Among all 3999 CI statements (DE + UK + US), 1072 (26.8%; 95% CI 25.5–28.2%) were rated as unclear from a prescriber perspective. The proportion of unclear statements was similar between the three drug markets (Germany: 27.2%; (95% CI 25.2–29.2%), UK: 28.5% (26.2–30.9%), US: 22.6% (19.7–25.8%)). However, the clarity varied notably between the different categories as well as between specific subcategories (Figure 4): Of the combined (DE + UK + US) individual CI statements 20.0% of the patient-related, 11.9% of the medication-related and 30.5% of the disease-related were of insufficient clarity. Focusing on the biggest category of disease-related CIs (summarized for DE/UK/US), 243 (7.3%) terms were related to the subcategory liver and 184 (75.7%) of them did not fulfill the criterion of sufficient clarity to a human prescriber, followed by nervous system (unclear: 54.1%) and laboratory discrepancies (unclear: 54.1%). On the other hand, the clearest terms were those associated with the subcategories immune system (unclear: 4.9%), skin (unclear: 5.9%) and neoplasia (unclear: 7.6%) (Figure 4). The relative distribution of clarity was very similar in all three countries (Figures S1 and S2). A more detailed analysis of the less frequent categories of medication- and patient-related CIs is shown in Figure S1.

### 3.5. Expressions of CIs

An exemplary overview of problematic CI statements is shown in Table 1. The three main factors leading to problematic expressions are listed in the Methods section chapter ‘Clarity’.

### 3.6. Codability

**Figure 5:**
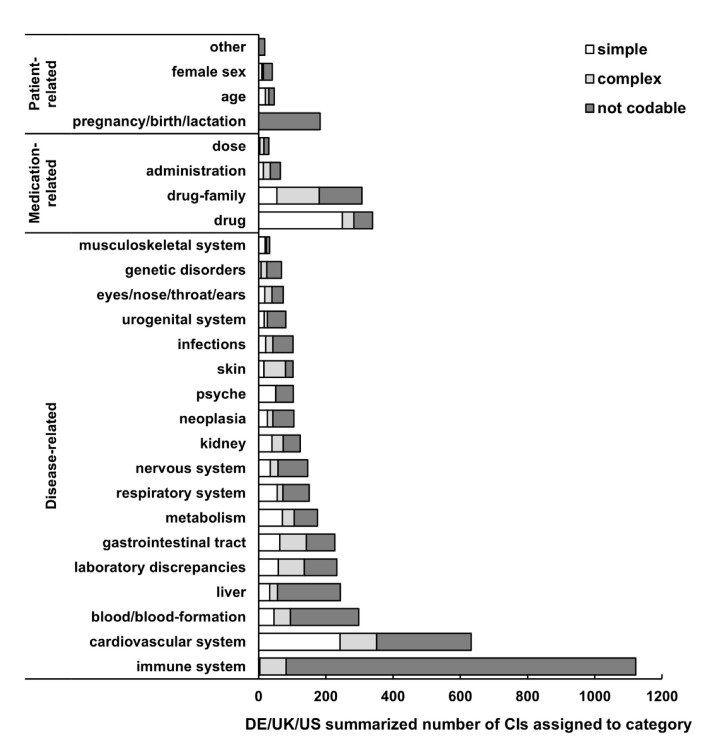
The average codability from the machine perspective of all absolute CIs in Germany and UK SmPCs and US PI is shown in total numbers of individual CIs assigned to each category, summarized for Germany, UK and US. The three categories patient-, medication- and disease-related CIs are sorted by frequency among all CIs, as well as the individual subcategories. White: simple codability, medium grey: complex codability, dark grey: not codable. CI: contraindication, DE: Germany.

Analogous to clarity, codability varied for each of the three categories and their subcategories (Figure 5) and, for DE/UK/US, an average of 62.5% (95% CI 61.0–64.0%) of the absolute CI statements were not codable. The results varied only slightly in between countries with a percentage of not codable CIs of 60.9% (95% CI 58.6–63.1%) for Germany, 63.6% (61.0–66.0%) for UK and 64.7% (61.2–68.1%) for US. Among the disease-related terms, in DE/UK/US average, the subcategories immune system (not codable: 92.7%), liver (not codable: 77.0%) and blood/blood formation (not codable: 68.1%) were the most problematic groups. The groups with the highest codability level were skin (not codable: 21.6%) musculoskeletal system (not codable: 30.3%) and gastrointestinal tract (not codable: 37.4%) in DE/UK/US average. The detailed analysis shown for each country can been seen in Figure S3 (disease-related CIs) and Figure S4 (medication- and patient-related CIs).

### 3.7. Inter-Rater Reliability

The evaluation of 400 CI items (10.0% of all 3999) by a second analyst showed an interrater agreement of 96.8% (Cohen’s ĸ = 0.93) for criteria ‘clarity’ and 95.5% (Cohen´s ĸ = 0.91) for criteria ‘codability’. According to the literature, this indicates ‘almost perfect’ agreement [32].

### 3.8. ICD Codability

Among all 3999 absolute CIs in DE/UK/US SmPCs/PI we found 3341 disease related expressions, of which 691 (20.7%) can fully or partially be described by one or more specific ICD-10 code(s). ‘Partially’ in this context means that the ICD-10 code specified one part of an expression, whilst an additional code category such as a drug ATC was required for the complete determination (e.g., ‘diabetes mellitus’ in term ‘contraindicated with aliskiren in patients with diabetes mellitus’). In the case of 394 (11.8%) of the disease-related CIs the ICD-10 code may be used as input for a more complex algorithm to determine the disease. Finally, 2215 (66.5%) of the disease-related terms could not be adequately described by an ICD-10 code at all. An exemplary overview of codable and not codable terms is shown in Table 2.

## 4. Discussion

This analysis of SmPCs/PI from three major drug markets indicates that a significant proportion of CI warnings may not be suitable to guide safe prescribing or electronic clinical decision support as intended. Moreover, there was a substantial variation of CI warnings for the same drugs across the different regulatory backgrounds. Regarding causes and consequences of these findings, the detailed classification of the CI warnings as well as the comparison of CI in different drug markets may offer some explanations.

The first parameter we analyzed in detail for all SmPCs/PI was ‘clarity’, which was an evaluation of the unambiguity of each CI statement from the prescriber perspective. The EMA stipulates on this topic in the SmPC guideline that a contraindicated clinical situation should be ‘unambiguously, comprehensively and clearly outlined’ [6]. Applying this definition, which broadly corresponds to the definition of clarity used in this analysis, we found that almost a third of the terms are unclear (Figure 4) with only slight differences in between the three countries. A more detailed analysis of the disease-related CIs revealed that, in addition to the frequently unclear liver-related CIs, the CIs related to the nervous system and laboratory discrepancies are those with the greatest ambiguity (Figure 4). In contrast to CIs related to clinical conditions, for medication-related and patient-related CIs a smaller proportion of unclear expressions was observed (DE/UK/US average: unclear: 11.9% and 20.0%, respectively), but these groups only accounted for a small fraction of all absolute CIs (Figure S1).

It should be considered that for certain clinical situations such as liver diseases or diseases of the cardiovascular system, different decision making by the prescribers, caused by different interpretation of the CIs, may lead to a highly heterogeneous drug therapy and thus to a possible endangerment of the patient’s well-being. Conversely, a too broad or clinically too conservative interpretation of CI may also lead to undertreatment (e.g., ‘is contraindicated in women who may become pregnant’).

Particularly in ‘problematic’ clinical situations, standardized decision making would be important for maintaining a high-quality standard of drug therapy [33,34]. We have found a high variability of expressions for many disease groups such as hematopoietic disorders, lung diseases or liver diseases (Table 1 and Table 2). We acknowledge that a SmPC/PI cannot address all possible clinical cases—i.e., complex clinical judgement can only partially be translated into simple terms and statements provided as absolute CI. However, the terms that are mentioned should at least be clear. Weersink et al. in 2019 observed, that a patient with liver cirrhosis, for example, would or would not fall under the definition of ‘hepatic impairment’ depending on the prescriber’s consideration, resulting in unequal drug treatment [13]. Salgado et al. came to similar conclusions in 2013 in relation to the information given in SmPCs/PI for dose adjustment in renal insufficiency [8].

During the initial analysis of the mere presence and number of absolute CIs in the SmPCs/PI, we have found some major differences between the three included markets, Germany, UK and US (Figure 2). While all German SmPCs listed absolute CI, 2.5% of UK and 10.1% of US SmPCs/PI listed no absolute CI (Figure 2A). Moreover, German SmPCs listed on average 11.7, while UK and US SmPCs/PI listed 9.0 and 4.6 absolute CIs on average, respectively (Figure 2B). The ‘Boxed Warning’ section, which is only present in US PI, was not included in the calculations. The presence of the ‘Boxed Warning’ sections and their content did not cover the previously mentioned gap in number of CIs (results not shown).

These discrepancies can partly be explained by different legal systems and regulatory requirements when comparing DE/UK with the US. The EMA demands a list of all situations within the CIs section, which can be based on either data or strong theoretical reasons, where the drug must not be given for safety reasons [6]. In contrary, FDA requirements state that CIs should be only for those clinical situations for which the risk from use of the drug clearly outweighs any possible therapeutic benefit and which are based on only on known hazards and not theoretical possibilities [17]. Especially regarding ‘hypersensitivity reactions’, there are strong regulatory discrepancies. Whilst EMA requires a general inclusion of the CI ‘hypersensitivity’, FDA only requires an inclusion and exemplary description of such, when there have been demonstrated cases and for which risk from use outweighs any benefits [6,17]. Apart from the regulatory differences between Europe and US, the differences between DE and UK cannot (yet) be explained by regulatory discrepancies, as the SmPCs are still based on the same EMA guidelines (disregarding the transition from EMA to MHRA (Medicines and Healthcare products Regulatory Agency) due to Brexit). Earlier studies (e.g., de Barros (2000) [33]: Brazil versus US; Alshammari (2017) [35]: US versus Canada and UK) have similarly shown, for sections such as indications, adverse events or dosing, that SmPCs/PI varied significantly when comparing countries with different as well as similar regulatory backgrounds, leading to a double standard in drug therapy [12,33,35].

The FDA clearly recommends precise language such as ‘is contraindicated’ instead of ‘should not be used’ [17]. Nevertheless, we found several CI sections in US PI with such a relative wording (e.g., PI for estradiol or levonogestrel/ethinylestradiol) [36,37]. However, we have also found relative CIs in UK SmPCs, where the CI sections started with a ‘should not’ expression, leading to an unclear statement (e.g., in UK SmPCs for aspirin or atenolol, there is no absolute CI), although the EMA guideline clearly specifies that in this section situations should be described in which the drug must not be given [6,38,39]. In contrast to the discrepancies in quantitative comparison, the CIs are relatively similar in terms of content (Figure 3).

The second perspective of our study was the ‘codability’ for use in CDSS, i.e., from a machine perspective [40]. In comparison to the earlier described clarity from the prescriber perspective, the codability on a simple or complex level was notably lower, with an average share of 37.5% of absolute CIs, which were not codable according to our definition (Figure 5, Figures S3 and S4). We may have used the term ‘code’ a little conservatively, for example, by classifying certain specific pharmacogenetic factors or epidemiological parameters as not codable, by which we intended to simulate the ability to map an expression by a ‘simple’ algorithm-based machine (non-artificial intelligence), with access to an ordinary electronic health record. This is also reflected in our results for patient-related CIs, which had the lowest level of codability at 15.6%, but also, as stated earlier, only represent a small amount (6.8%) of all absolute CIs. However, since we have also included ‘hand-programmed algorithms’ to define CIs as codable on a complex level in addition to simple codes such as ICD or ATC, our definition of codability should nevertheless have reflected the current state of digitalization in the healthcare system relatively well. In terms of codability from a machine perspective, the disease-related CIs, which represented the most absolute among all analyzed SmPCs/PI (83.5%), had the lowest codability with 66.2% of CIs not codable on average. These results underline that an uncomplicated, unambiguous and comprehensive implementation of text-based CIs into an electronic system, which can map these CIs to a standardized code system, is currently not feasible.

The ICD-10 code is an internationally widely used system to classify diseases, which is why we used it to map disease-related CIs. In our analysis, we found that 66.5% of the disease-related CIs could not be represented fully or partially (as part of an algorithm) by using ICD-10 codes. As shown in Table 2, depending on the SmPC/PI analyzed, there were both good and clear expressions that could be mapped into ICD-10 codes, as well as corresponding negative examples which could not be mapped with certainty to a definite set of ICD terms. These examples show that it was not the code system itself lacking quality (which certainly also has limits) but the means of expression in the SmPC/PI that was responsible for the impossibility of digital mapping. The use of vague expressions may even be intended by the authors of the SmPC/PI (ergo, the pharmaceutical companies) to transfer any legal responsibility and decision-making regarding a CI to the prescriber by using the widest possible expression with room for interpretation, according to the motto ‘to use a sledge-hammer to crack a nut’.

It should also be emphasized that the ICD-10 system currently may predominantly be used for cause of death reporting and billing in the medical sector and not as a tool for the clinically precise documentation of diseases and procedures. The current ICD-10 system clearly has its limits when different clinical diagnoses are summarized within one code, and therefore cannot be distinguished from each other with this system and consequently mapped digitally. This ‘weakness’ will hopefully be reduced or eliminated with the implementation of the new ICD-11 code (started in January 2022), since new codes and a new coding procedure were introduced here (e.g., for ‘pregnancy’ or ‘hypersensitivity’), but this was not the focus of this work [41,42]. However, many ambiguous expressions describing absolute CIs that we found in our analysis would also not be better mapped to the ICD-11 code, because the choice of words of many CIs was vague itself and remains ambiguous in terms of codability. In a study by Seidling et al. in 2010, it was discussed that there were both advantages and disadvantages in coded entry versus free text entry [43]. As part of our work with the expressions in CIs, however, we were able to show that it is possible to put a clearly understandable and at the same time ‘well-codable’ expression text in an absolute CI (Table 2, first column) and that these two attributes did not conflict with each other.

## 5. Conclusions

Many absolute CIs in SmPCs/PI are either not sufficiently clear for the prescriber and/or not interpretable by a computerized system. This may pose an unnecessary risk for medication safety. More quality control with respect to clinical and technical usability of SMPCs/PI as well as efforts for their international harmonization are needed to ensure that each user (man or machine) worldwide comes to the same decision.

Pharmaceutical companies and other stakeholders should find a standardized and harmonized approach to create unambiguous and precisely expressed CIs to avoid vague terminology and the same applies to the regulatory authorities during the review and approval of the SmPCs/PI. One possibility would be to roll out an internationally, freely available, evidence-based tool with standardized terminology to create a uniform quality standard worldwide. Commercial databases such as ‘UpToDate’, which already provide distinct SmPC/PI information for pediatrics and adults, can serve as a model here [44].

The critical content of SmPCs/PI should routinely be subjected to pretests in the intended audience regarding comprehensibility. As a first step, we recently initiated a survey of physicians and pharmacists regarding their understanding of very common CI terms. Furthermore, medication errors and adverse drug events observed as routine should be investigated more thoroughly regarding a possible relation to unclear wording of SmPCs/PI.

## Figures and Tables

**Table 1:** Examples for problematic expressions in contraindications of SmPCs/PI that may leave too much room for interpretation or lack clear cut-off so that different prescribers may arrive at different conclusions as to whether the condition of a CI is fulfilled in a patient or not, or as to which internationally agreed reference lists are missing. Each ambiguous term is underlined.

Country	Drug (SmPC/PI)	Expression in SmPC/PI
DE/UK/US	Alendronic acid (Fosamax)	abnormalities to the esophagus which delay esophageal emptying
DE	Amisulpride (Solian)	drugs that can trigger serious cardiac arrhythmias
DE/UK	Amlodipine (Norvasc, ISTIN)	high grade aortic stenosis
DE/UK	Apixaban (Eliquis)	recent spinal surgery
DE/UK	Apixaban (Eliquis)	clinical situation if considered a significant risk factor for major bleeding
DE	Aspirin (Aspirin)	previous hypersensitivity after taking salicylates or substances having a similar effect, particularly NSAIDs
DE	Bisoprolol (Concor)	late stages of peripheral arterial occlusive disease
DE/UK	Bupropion (Elontril, Zyban)	patient undergoing abrupt discontinuation from any medicinal product known to be associated with risk of seizures on withdrawal
DE/UK	Buspiron (Anxut, generic)	intoxication with antipsychotic drugs
US	Carvedilol (Coreg)	severe bradycardia
DE	Chlorthalidone (Hygroton)	conditions with increased potassium losses
DE/UK/US	Clopidogrel (Plavix)	active pathological bleeding
DE	Codeine phosphate/paracetamol (Gelonida)	approaching birth
UK	Digoxin (Lanoxin)	AV bock second degree, especially if there is a history of Stokes-Adams attacks
US	Ethinylestradiol/norgestimate (generic)	in women: thrombogenic valvular disease
US	Ethinylestradiol/norgestimate (generic)	in women: uncontrolled hypertension
DE/UK/US	Fenofibrate (Lipidil, Supralip, Tricor)	unexplained (only US/UK) persistent liver function abnormalities
DE/UK	Finasteride (Proscar)	children
UK	Folic acid (generic)	malignant diseases unless megaloblastic anemia due to folic acid deficiency
DE/US	Levothyroxine sodium (Euthyrox, Unithroid)	untreated (only DE)/uncorrected (only UK) adrenal insufficiency
DE/UK	Lisdexamfetamine (Elvanse)	advanced arteriosclerosis
US	Metformin/sitagliptin (Janumet)	chronic diabetic ketoacidosis
DE	Methotrexate (Lantarel)	increased alcohol consumption
DE/UK	Methylphenidate (Ritalin)	potentially life-threatening arrhythmia
US	Metoprolol (Lopressor)	moderate cardiac failure
US	Morphine (Duramorph), Oxycodone (Roxicodone), Tramadol (Ultram)	significant respiratory depression
US	Opicapone (Ongentys)	catecholamine-secreting neoplasms
UK	Oxycodone (Lynlor)	elevated carbon dioxide levels in the blood
DE	Propranolol (Dociton)	higher grade sino-atrial block
UK	Metoprolol (generic)	
UK	Rosuvastatin (Crestor)	for 40 mg dosis: situations where an increase in plasma levels may occur
DE/UK	Telmisartan (Micardis)	biliary obstructive disorders
US	Trimethoprim/sulfamethoxazole (Bactrim)	marked hepatic damage
DE/UK/US	Valproic acid (Orfiril, Convulex, Depakene)	women of childbearing potential
US	Warfarin (Coumadin)	major regional anesthesia
US	Warfarin (Coumadin)	traumatic surgery resulting in large open surfaces

CI: contraindication, DE: Germany, NSAID: nonsteroidal anti-inflammatory drug, PI: Prescribing Information, SmPC: Summary of Product Characteristics.

**Table 2:** Exemplary expressions in CIs: both codable expressions and their ICD-10 codability and thematically related not codable expressions are shown.

Codable Terms			Not Codable Terms
Expression in SmPC/PI	ICD-10	ICD-10 Description	Expression in SmPC/PI
Breast cancer	C50	Malignant neoplasm of breast	Estrogen-dependent tumor
			Sex hormone dependent malignant tumors
Diseases of the hematopoietic system	D50-D77	Diseases of the blood and blood-forming organs	Condition if considered a significant risk factor for major bleeding
			Preexisting blood dyscrasias
Hemorrhagic diathesis	D69	Purpura and other hemorrhagic conditions	Blood clotting disorders
			Thrombophilic disorders
Sleep apnea syndrome	G47.3	Sleep apnea	Complex sleep behaviour
			Unusual sleep behaviour
Myasthenia gravis	G70.0	Myasthenia gravis	Patients with predisposing factors for myopathy
Esophageal varices	I85	Esophageal varices	Diseases of the esophagus which delay esophageal emptying
Asthma	J45	Asthma	Reactive airway disease
			Respiratory disease with bronchospastic component
			Bronchial hyperreactivity
Crohn´s disease	K50	Crohn disease (regional enteritis)	Severe intestinal inflammation associated with symptomatic stenosis
Paralytic ileus	K56.0	Paralytic ileus	Delayed gastric emptying
Primary biliary cirrhosis	K74.3	Primary biliary cirrhosis	Severe hepatic impairment
			Hepatic diseases
			Hepatic dysfunction
			liver parenchymal damage
			Hepatic diseases associated with coagulopathy and clinically relevant bleeding risk
Gout	M10	Gout	Symptomatic hyperuricemia
Acute abdomen	R10.0	Acute abdomen	Abdominal pain of unknown origin

CI: contraindication, ICD: International Statistical Classification of Diseases and Related Health Problems, PI: Prescribing Information, SmPC: Summary of Product Characteristics.

## Supplementary Materials

The following supporting information can be downloaded at: https://www.mdpi.com/article/10.3390/jcm11144167/s1. Figure S1: Clarity of patient- and medication-related CIs by country; Figure S2: Clarity of disease-related CIs by country; Figure S3: Codability of disease-related CIs by country; Figure S4: Codability of patient- and medication-related CIs by country; Table S1: List of 158 German Summaries of Product Characteristics (SmPCs) included in the analysis; Table S2: List of 158 UK Summaries of Product Characteristics (SmPCs) included in the analysis; Table S3: List of 158 US Prescribing Information (PI) included in the analysis; Table S4: Extraction and evaluation of CIs for example rivaroxaban (Xarelto) UK SmPC.
