# Supplementary material for: Comprehensibility of Contraindications in German, UK and US Summaries of Product Characteristics/Prescribing Information—A Comparative Qualitative and Quantitative Analysis
Source: J Clin Med. 2022 Jul 18;11(14):4167. doi: 10.3390/jcm11144167 (PMC9316253; doi:10.3390/jcm11144167)

Supplemental Figure S1. Clarity of patient- and medication-related CIs by country. The clarity from the prescriber perspective of all medication-related (A) and patient-related (B) absolute CIs in DE and UK SmPCs and US PI is shown in absolute numbers of individual CIs assigned to each category. The categories are sorted by frequency among all CIs. White: clear, grey: unclear. CI: contraindication, DE: German, PI: Prescribing Information, SmPC: Summary of Product Characteristics

A.

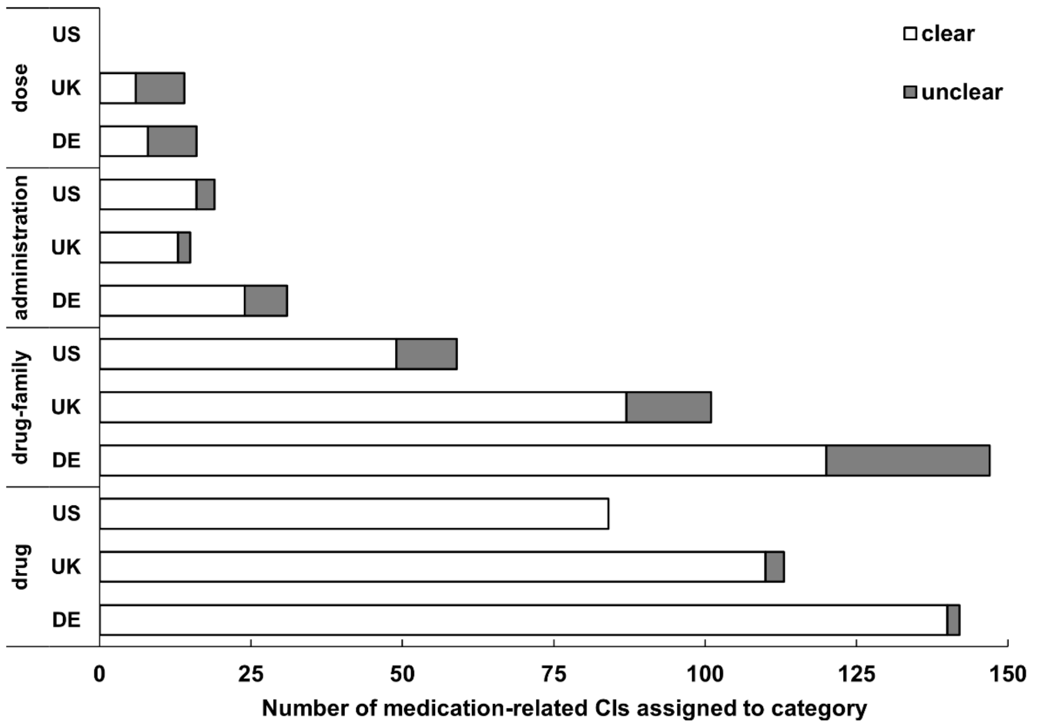

B.

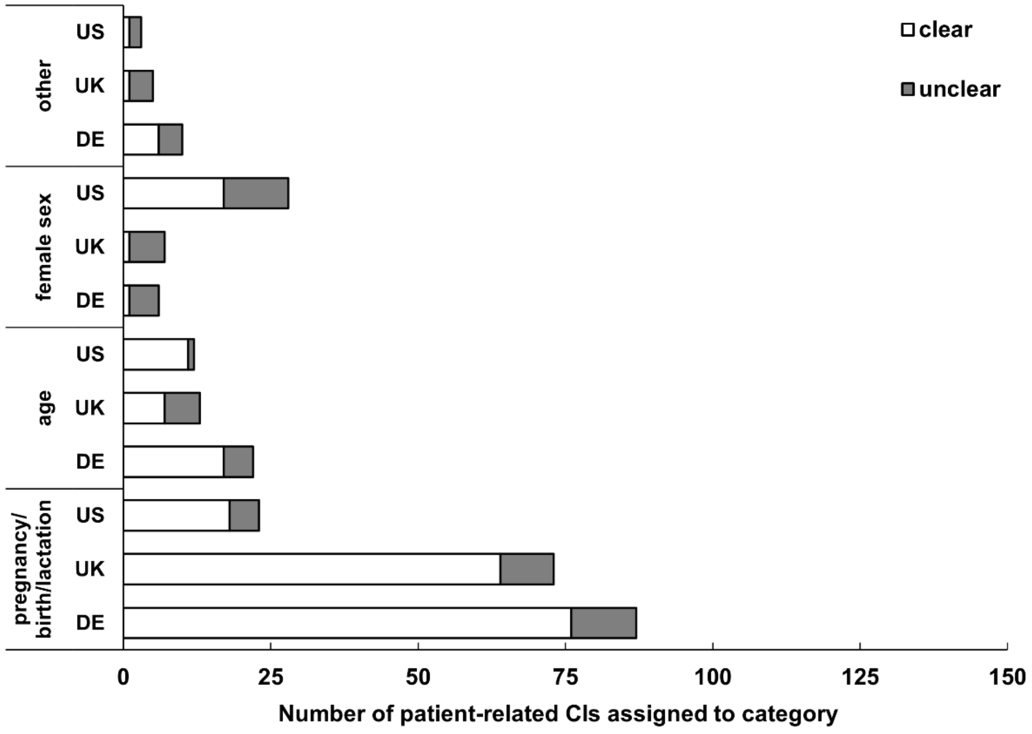

Supplemental Figure S2. Clarity of disease-related CIs by country. The clarity from the prescriber perspective of all disease-related absolute CIs in DE and UK SmPCs and US PI is shown in absolute numbers of individual CIs assigned to each category. The categories are sorted by frequency among all CIs. White: clear, grey: unclear. CI: contraindication, DE: German, PI: Prescribing Information, SmPC: Summary of Product Characteristics

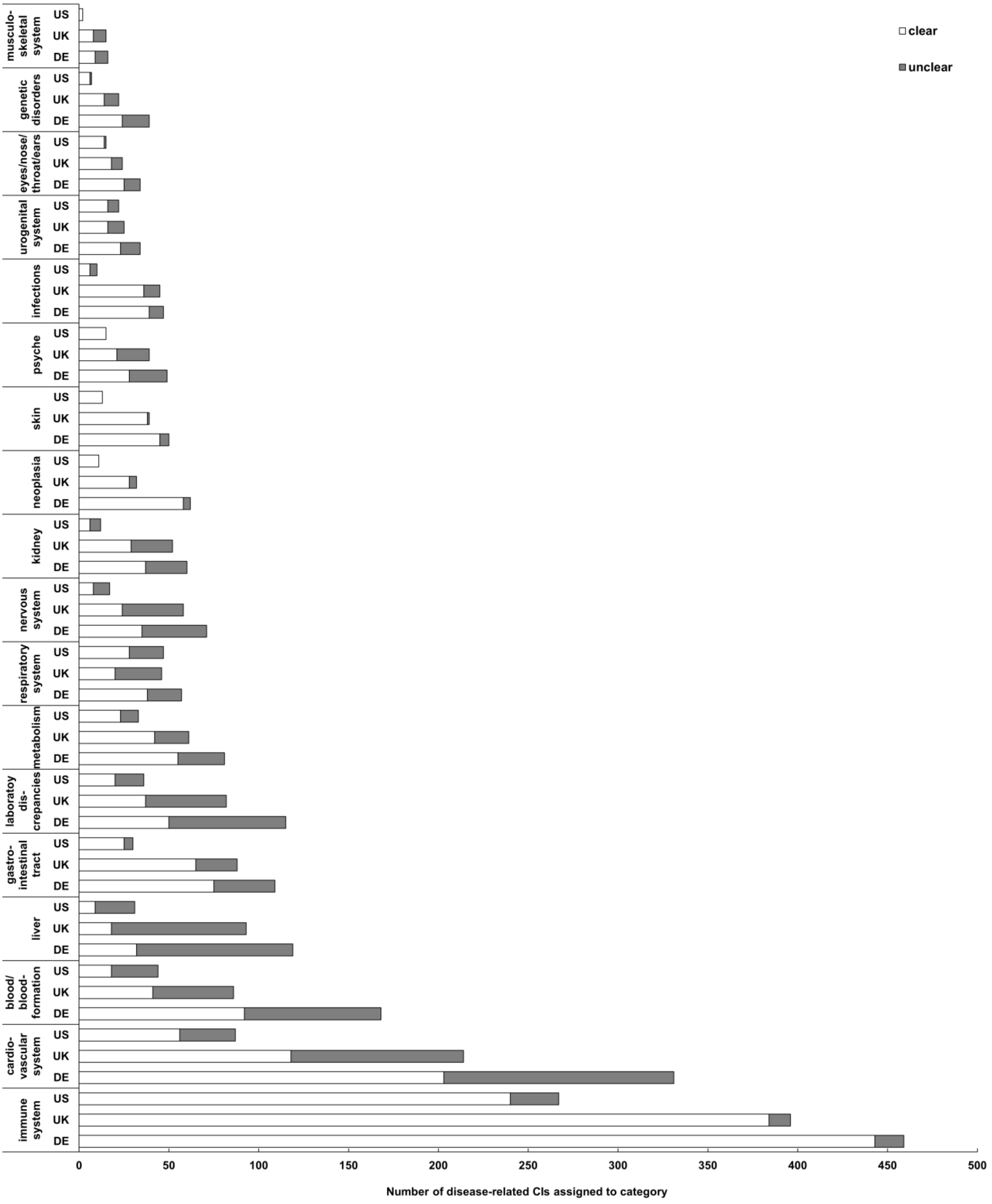

Supplemental Figure S3. Codability of disease-related CIs by country. The codability from the machine perspective of all disease-related absolute CIs in DE and UK SmPCs and US PI is shown in absolute numbers of individual CIs assigned to each category. The categories are sorted by frequency among all CIs. White: simple codability, medium grey: complex codability, dark grey: not codable. CI: contraindication, DE: German, PI: Prescribing Information, SmPC: Summary of Product Characteristics

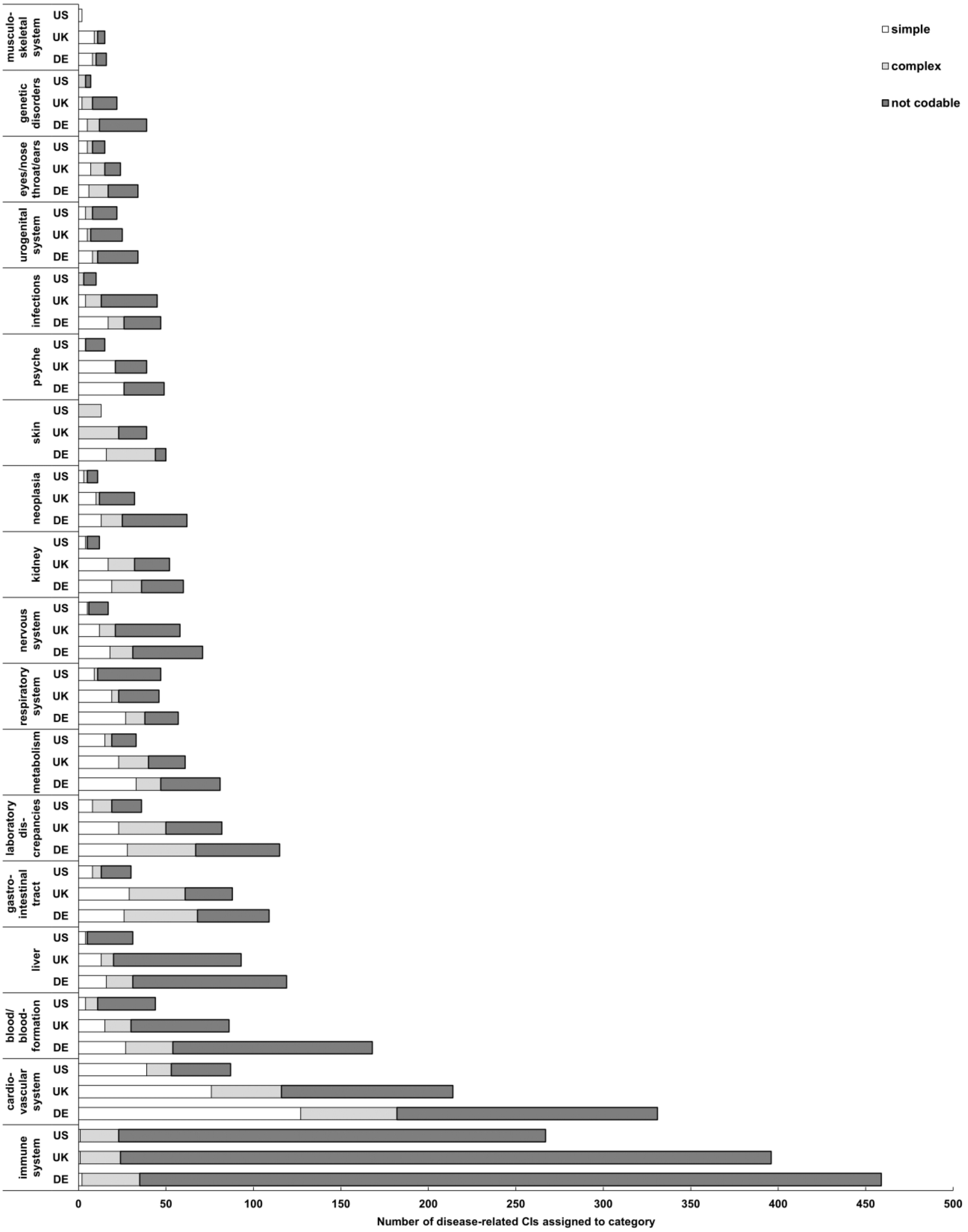

Supplemental Figure S4. Codability of patient- and medication-related CIs by country. The codability from the machine perspective of all medication-related (A) and patient-related (B) absolute CIs in DE and UK SmPCs and US PI is shown in absolute numbers of individual CIs assigned to each category. The categories are sorted by frequency among all CIs. White: simple codability, medium grey: complex codability, dark grey: not codable. CI: contraindication, DE: German, PI: Prescribing Information, SmPC: Summary of Product Characteristics

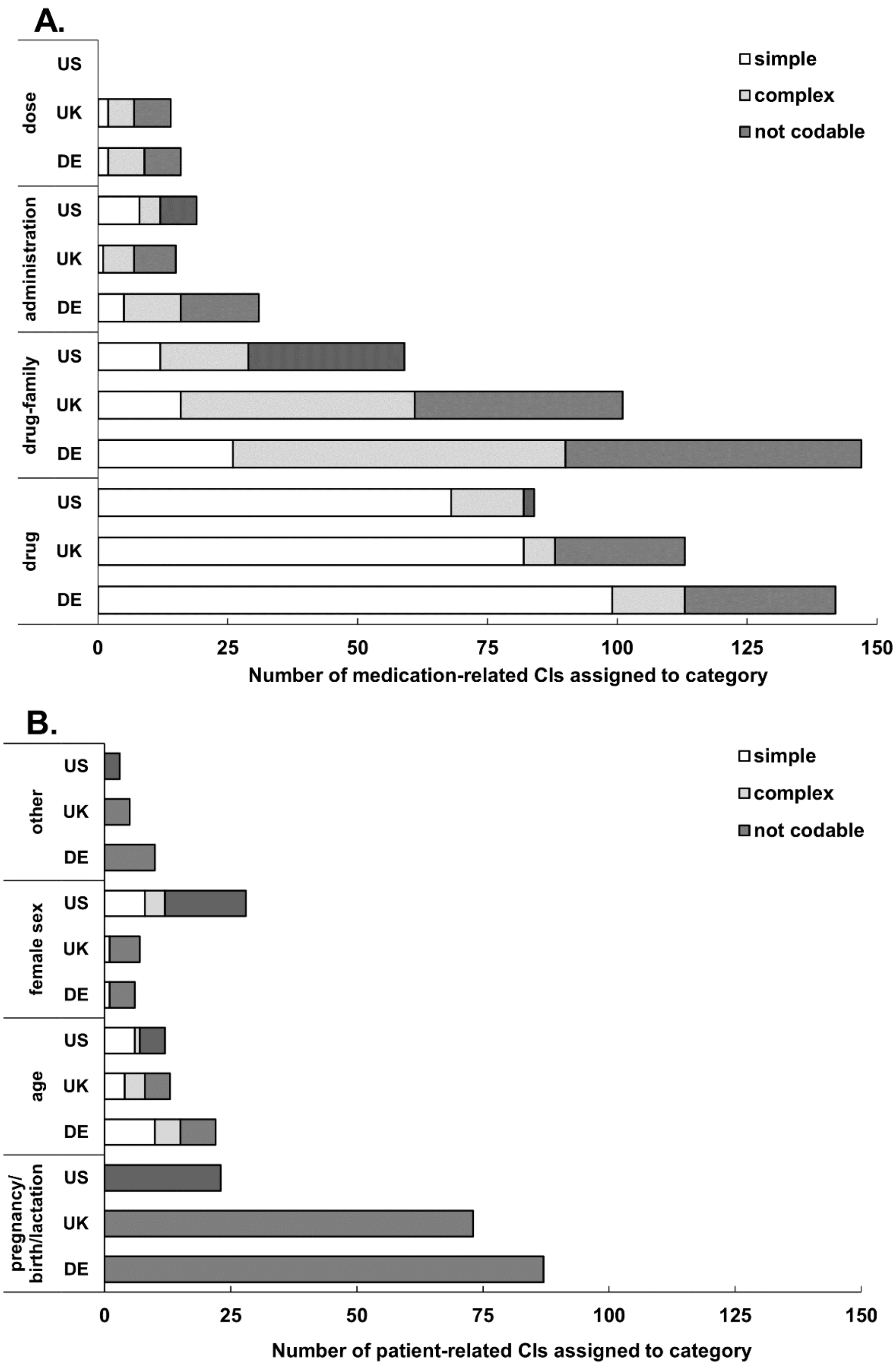

Supplement: Supplementary file 1 [file jcm-11-04167-s001.zip › Supplemental Figures_20220718_clean.pdf]
