# Supplementary material for: Comprehensibility of Contraindications in German, UK and US Summaries of Product Characteristics/Prescribing Information—A Comparative Qualitative and Quantitative Analysis
Source: J Clin Med. 2022 Jul 18;11(14):4167. doi: 10.3390/jcm11144167 (PMC9316253; doi:10.3390/jcm11144167)
Supplement: Supplementary file 1 [file jcm-11-04167-s001.zip › Supplemental Table S1_20220310.pdf]

Supplemental Table S1. List of 158 German Summaries of Product Characteristics (SmPCs) included in the analysis

Drug(s)	SmPC	Manufacturer	SmPC update	URL	Access date
Alendronic acid	Fosamax®	Organon	Feb 2021	https://www.fachinfo.de/api/fachinfo/pdf/000018	11 Aug 2021
Allopurinol	Allopurinol-ratiopharm®	Ratiopharm	Nov 2018	https://www.fachinfo.de/api/fachinfo/pdf/000073	11 Aug 2021
Alprazolam	Tafil®	Pfizer	Jul 2020	https://www.fachinfo.de/api/fachinfo/pdf/002008	11 Aug 2021
Amisulpride	Solian®	Sanofi	Apr 2019	https://www.fachinfo.de/api/fachinfo/pdf/004729	12 Aug 2021
Amitriptyline	Syneudon®	Krewel Meuselbach	Nov 2018	https://www.fachinfo.de/api/fachinfo/pdf/006568	11 Aug 2021
Amlodipine	Norvasc®	Pfizer	Jun 2020	https://www.fachinfo.de/api/fachinfo/pdf/007631	11 Aug 2021
Amoxicillin	Amoxicillin-ratiopharm®	Ratiopharm	Jan 2021	https://www.fachinfo.de/api/fachinfo/pdf/020886	11 Aug 2021
Amoxicillin/clavulanic acid	Augmentan	GlaxoSmithKline	Jun 2020	https://www.fachinfo.de/api/fachinfo/pdf/006193	11 Aug 2021
Apixaban	Eliquis®	Bristol Myers Squibb / Pfizer	Apr 2021	https://www.fachinfo.de/api/fachinfo/pdf/021995	11 Aug 2021
Aspirin	Aspirin®	Bayer	Oct 2018	https://www.fachinfo.de/api/fachinfo/pdf/000174	11 Aug 2021
Atenolol	Atenolol-ratiopharm®	Ratiopharm	Feb 2021	https://www.fachinfo.de/api/fachinfo/pdf/000184	11 Aug 2021
Atorvastatin	Sortis®	Pfizer	Jun 2021	https://www.fachinfo.de/api/fachinfo/pdf/000291	11 Aug 2021
Azithromycin	Zithromax®	Pfizer	May 2021	https://www.fachinfo.de/api/fachinfo/pdf/015933	11 Aug 2021
Beclometasone	Ventolair®	Teva	Mar 2021	https://www.fachinfo.de/api/fachinfo/pdf/009776	11 Aug 2021
Belantamab mafodotin	Blenrep	GlaxoSmithKline	Jun 2021	https://www.fachinfo.de/api/fachinfo/pdf/023119	12 Aug 2021
Bempedoic acid	Nilemdo	Daiichi Sankyo	Dec 2020	https://www.gelbe-liste.de/produkte/Nilemdo-180-mg-Filmtabletten_1273210/fachinformation	23 Aug 2021
Betamethasone	Celestan®	Organon	Mar 2021	https://www.fachinfo.de/api/fachinfo/pdf/003053	11 Aug 2021
Bisoprolol	Concor	Merck	Nov 2020	https://www.fachinfo.de/api/fachinfo/pdf/003991	12 Aug 2021
Botulinum toxin type A	Botox®	Allergan	Dec 2020	https://www.fachinfo.de/api/fachinfo/pdf/020408	11 Aug 2021
Brolucizumab	Beovu®	Novartis Pharma	Sep 2020	https://www.fachinfo.de/api/fachinfo/pdf/022909	12 Aug 2021
Budesonide	Pulmicort® Turbohaler®	AstraZeneca	Aug 2018	https://www.fachinfo.de/api/fachinfo/pdf/005511	11 Aug 2021
Bupropion	Elontril	GlaxoSmithKline	Mar 2021	https://www.fachinfo.de/api/fachinfo/pdf/010164	11 Aug 2021
Buspirone	Anxut®	Eisai	May 2020	https://www.fachinfo.de/api/fachinfo/pdf/003411	11 Aug 2021
Candesartan	Candecor®	TAD Pharma	Sep 2020	https://www.fachinfo.de/api/fachinfo/pdf/023026	11 Aug 2021
Carvedilol	Querto®	Takeda	Feb 2015	https://www.fachinfo.de/api/fachinfo/pdf/007690	11 Aug 2021

Drug(s)	SmPC	Manufacturer	SmPC update	URL	Access date
Cefalexin	Cephalexin-ratiopharm®	Ratiopharm	Nov 2019	https://www.fachinfo.de/api/fachinfo/pdf/003175	11 Aug 2021
Cefiderocol	Fetcroja	Shionogi	Apr 2020	https://www.fachinfo.de/api/fachinfo/pdf/023073	12 Aug 2021
Chlortalidone	Hygroton®	Trommsdorff	Sep 2020	https://www.fachinfo.de/api/fachinfo/pdf/005243	11 Aug 2021
Citalopram	Cipramil®	Lundbeck	Nov 2020	https://www.fachinfo.de/api/fachinfo/pdf/002148	11 Aug 2021
Clonazepam	Antelepsin®	Desitin	Jun 2020	https://www.fachinfo.de/api/fachinfo/pdf/000376	11 Aug 2021
Clonidine	Catapresan®	Glenwood	Aug 2020	https://www.fachinfo.de/api/fachinfo/pdf/000355	11 Aug 2021
Clopidogrel	Plavix®	Sanofi	May 2021	https://www.fachinfo.de/api/fachinfo/pdf/003345	11 Aug 2021
Codeine phosphate/paracetamol	Gelonida®	Pfizer	May 2021	https://www.fachinfo.de/api/fachinfo/pdf/007142	11 Aug 2021
Cyanocobalamin	Vitamin B_{12}-ratiopharm®	Ratiopharm	Jul 2015	https://www.fachinfo.de/api/fachinfo/pdf/002290	11 Aug 2021
Dexamethasone	Dexamethason-ratiopharm®	Ratiopharm	Dec 2020	https://www.fachinfo.de/api/fachinfo/pdf/008836	12 Aug 2021
Diazepam	Diazepam-ratiopharm®	Ratiopharm	Apr 2018	https://www.fachinfo.de/api/fachinfo/pdf/011210	11 Aug 2021
Diclofenac	Voltaren Dolo	GlaxoSmithKline	Nov 2019	https://www.fachinfo.de/api/fachinfo/pdf/022158	11 Aug 2021
Digoxin	Lenoxin®	Aspen	Sep 2019	https://www.fachinfo.de/api/fachinfo/pdf/001548	12 Aug 2021
Diltiazem	Dilzem®	Pfizer	May 2021	https://www.fachinfo.de/api/fachinfo/pdf/005414	11 Aug 2021
Dostarlimab	Jemperli	GlaxoSmithKline	Jul 2021	https://www.fachinfo.de/api/fachinfo/pdf/023363	12 Aug 2021
Doxazosin	Cardular®	Pfizer	May 2020	https://www.fachinfo.de/api/fachinfo/pdf/008724	11 Aug 2021
Doxycycline	Oraycea	Galderma	Apr 2021	https://www.fachinfo.de/api/fachinfo/pdf/011791	11 Aug 2021
Duloxetine	Cymbalta®	Lilly	Jun 2020	https://www.fachinfo.de/api/fachinfo/pdf/008807	11 Aug 2021
Edoxaban	Lixiana®	Daiichi Sankyo	Nov 2020	https://www.gelbe-liste.de/produkte/Lixiana-60-mg-Filmtabletten_891971/fachinformation	23 Aug 2021
Elexacaftor/ivacaftor/tezacaftor	Kaftrio	Vertex	Apr 2021	https://www.fachinfo.de/api/fachinfo/pdf/023110	12 Aug 2021
Empagliflozin	Jardiance®	Boehringer Ingelheim / Lilly	Jun 2021	https://www.fachinfo.de/api/fachinfo/pdf/021054	11 Aug 2021
Enalapril	Xanef®	Organon	Jun 2021	https://www.fachinfo.de/api/fachinfo/pdf/002315	11 Aug 2021
Enoxaparin sodium	Clexane®	Sanofi	Apr 2020	https://www.fachinfo.de/api/fachinfo/pdf/021516	11 Aug 2021
Escitalopram	Cipralex®	Lundbeck	Nov 2020	https://www.fachinfo.de/api/fachinfo/pdf/005936	11 Aug 2021
Esomeprazole	Nexium®	Grünenthal	Feb 2020	https://www.fachinfo.de/api/fachinfo/pdf/008312	11 Aug 2021
Estradiol	Estrifam®	Novo Nordisk	Sep 2020	https://www.fachinfo.de/api/fachinfo/pdf/002180	11 Aug 2021

Drug(s)	SmPC	Manufacturer	SmPC update	URL	Access date
Ethinylestradiol/norethisterone	Conceplan® M	Pfizer	May 2021	https://www.fachinfo.de/api/fachinfo/pdf/002874	11 Aug 2021
Ethinylestradiol/norgestimate	Amicette	Aristo Pharma	Jun 2021	https://www.gelbe-liste.de/produkte/Amicette-250-Mikrogramm-35-Mikrogramm-Tabletten_823077/fachinformation	23 Aug 2021
Ezetimibe	Ezetrol®	Organon	Feb 2021	https://www.fachinfo.de/api/fachinfo/pdf/008015	11 Aug 2021
Felodipine	Modip®	AstraZeneca	Feb 2021	https://www.fachinfo.de/api/fachinfo/pdf/004467	11 Aug 2021
Fenofibrate	Lipidil®	Mylan	Sep 2020	https://www.fachinfo.de/api/fachinfo/pdf/004988	11 Aug 2021
Fexofenadine	Telfast®	Sanofi	May 2021	https://www.fachinfo.de/api/fachinfo/pdf/002236	11 Aug 2021
Finasteride	Proscar®	Organon	Feb 2021	https://www.fachinfo.de/api/fachinfo/pdf/006661	11 Aug 2021
Fluoxetine	Fluoxetin-ratiopharm®	Ratiopharm	Sep 2020	https://www.fachinfo.de/api/fachinfo/pdf/007815	11 Aug 2021
Fluticasone	Avamys	GlaxoSmithKline	Aug 2020	https://www.fachinfo.de/api/fachinfo/pdf/010709	11 Aug 2021
Fluticasone/salmeterol	Viani Diskus	GlaxoSmithKline	Aug 2020	https://www.fachinfo.de/api/fachinfo/pdf/005172	11 Aug 2021
Folic acid	DreisaFol®	Teva	Sep 2016	https://www.fachinfo.de/api/fachinfo/pdf/003127	11 Aug 2021
Formoterol	Foradil®	Novartis Pharma	Dec 2020	https://www.fachinfo.de/api/fachinfo/pdf/001143	11 Aug 2021
Formoterol/budesonide	Symbicort®Turbohaler®	AstraZeneca	Oct 2020	https://www.fachinfo.de/api/fachinfo/pdf/007354	11 Aug 2021
Furosemide	Lasix®	Sanofi	Sep 2017	https://www.fachinfo.de/api/fachinfo/pdf/005673	11 Aug 2021
Gabapentin	Neurontin®	Pfizer	Jul 2021	https://www.fachinfo.de/api/fachinfo/pdf/011567	11 Aug 2021
Glimepiride	Amaryl®	Sanofi	Apr 2017	https://www.fachinfo.de/api/fachinfo/pdf/007730	11 Aug 2021
Hydrocortisone	Hydrocortison 10 mg JENAPHARM®	Mibe	Aug 2020	https://www.fachinfo.de/api/fachinfo/pdf/011234	11 Aug 2021
Hydroxocobalamin	Depogamma®	Wörwag Pharma	Jul 2020	https://www.fachinfo.de/api/fachinfo/pdf/022776	11 Aug 2021
Ibuprofen	IBU-ratiopharm®	Ratiopharm	Jan 2021	https://www.fachinfo.de/api/fachinfo/pdf/014937	11 Aug 2021
Indapamide	Natrilix®	Servier Deutschland	Apr 2021	https://www.fachinfo.de/api/fachinfo/pdf/001428	11 Aug 2021
Insulin aspart	NovoRapid®	Novo Nordisk	Sep 2020	https://www.fachinfo.de/api/fachinfo/pdf/006754	11 Aug 2021
Insulin glargine	Lantus®	Sanofi	Jul 2020	https://www.fachinfo.de/api/fachinfo/pdf/007998	11 Aug 2021
Insulin human	Insuman®	Sanofi	Jul 2020	https://www.fachinfo.de/api/fachinfo/pdf/000373	11 Aug 2021
Insulin lispro	Humalog®	Lilly	Sep 2020	https://www.fachinfo.de/api/fachinfo/pdf/007637	11 Aug 2021
Irbesartan	Aprovel®	Sanofi	Mar 2021	https://www.fachinfo.de/api/fachinfo/pdf/001666	11 Aug 2021
Isatuximab	Sarclisa®	Sanofi Genzyme	Jun 2021	https://www.fachinfo.de/api/fachinfo/pdf/022995	12 Aug 2021

Drug(s)	SmPC	Manufacturer	SmPC update	URL	Access date
Isosorbide mononitrate	Ismo®	Riemser	Dec 2018	https://www.fachinfo.de/api/fachinfo/pdf/002406	11 Aug 2021
Lactulose	Bifiteral®	Mylan	Jul 2020	https://www.fachinfo.de/api/fachinfo/pdf/009521	11 Aug 2021
Lamotrigine	Lamictal	GlaxoSmithKline	Jan 2021	https://www.fachinfo.de/api/fachinfo/pdf/007443	11 Aug 2021
Lansoprazole	Agopton®	Takeda	Jan 2021	https://www.fachinfo.de/api/fachinfo/pdf/005438	11 Aug 2021
Latanoprost	Xalatan®	Pfizer	May 2020	https://www.fachinfo.de/api/fachinfo/pdf/001460	11 Aug 2021
Levetiracetam	Keppra®	UCB	Dec 2020	https://www.fachinfo.de/api/fachinfo/pdf/012886	11 Aug 2021
Levonogestrel/ethinylestradiol	Femikadin®	Besins Healthcare	Jun 2021	https://www.fachinfo.de/api/fachinfo/pdf/013769	11 Aug 2021
Levothyroxine sodium	Euthyrox®	Merck	Apr 2020	https://www.fachinfo.de/api/fachinfo/pdf/000811	11 Aug 2021
Lisdexamfetamine	Elvanse	Takeda	Jan 2021	https://www.fachinfo.de/api/fachinfo/pdf/020737	11 Aug 2021
Lisinopril	Lisinopril-TEVA®	Teva	Dec 2020	https://www.fachinfo.de/api/fachinfo/pdf/008146	11 Aug 2021
Lisinopril/hydrochlorothiazide	Lisinopril-ratiopharm® comp.	Ratiopharm	May 2020	https://www.fachinfo.de/api/fachinfo/pdf/008292	11 Aug 2021
Loratadine	Loratadin-ratiopharm	Ratiopharm	Jul 2018	https://www.fachinfo.de/api/fachinfo/pdf/003703	11 Aug 2021
Lorazepam	Tavor®	Pfizer	May 2021	https://www.fachinfo.de/api/fachinfo/pdf/002029	11 Aug 2021
Losartan	Lorzaar®	Organon	Feb 2021	https://www.fachinfo.de/api/fachinfo/pdf/008493	11 Aug 2021
Losartan/hydrochlorothiazide	Lorzaar® Plus	Organon	Feb 2021	https://www.fachinfo.de/api/fachinfo/pdf/011464	11 Aug 2021
Macrogol	Laxofalk®	Dr. Falk Pharma	Oct 2020	https://www.fachinfo.de/api/fachinfo/pdf/007246	11 Aug 2021
Meloxicam	Mobec®	Boehringer Ingelheim	Jan 2021	https://www.fachinfo.de/api/fachinfo/pdf/007736	11 Aug 2021
Mesalazine	Pentasa	Ferring Arzneimittel	Jan 2021	https://www.fachinfo.de/api/fachinfo/pdf/005332	11 Aug 2021
Metformin	Glucophage®	Merck	Oct 2020	https://www.fachinfo.de/api/fachinfo/pdf/000959	11 Aug 2021
Metformin/sitagliptin	Janumet®	MSD	Sep 2020	https://www.fachinfo.de/api/fachinfo/pdf/011094	11 Aug 2021
Methotrexate	Lantarel®	Pfizer	May 2021	https://www.fachinfo.de/api/fachinfo/pdf/004477	11 Aug 2021
Methylphenidate	Ritalin®	Novartis Pharma	Sep 2020	https://www.fachinfo.de/api/fachinfo/pdf/002958	11 Aug 2021
Metoprolol	Beloc-Zok®	Recordati Pharma	Oct 2020	https://www.fachinfo.de/api/fachinfo/pdf/003404	11 Aug 2021
Mirtazapine	Remergil SolTab®	Organon	Apr 2021	https://www.fachinfo.de/api/fachinfo/pdf/006081	11 Aug 2021
Mometasone	Ecural®	Organon	Feb 2021	https://www.fachinfo.de/api/fachinfo/pdf/007691	11 Aug 2021
Montelukast	Singulair®	Organon	Feb 2021	https://www.fachinfo.de/suche/fi/002942	11 Aug 2021
Morphine	Oramorph®	Kyowa Kirin	Nov 2020	https://www.fachinfo.de/api/fachinfo/pdf/010943	11 Aug 2021

Drug(s)	SmPC	Manufacturer	SmPC update	URL	Access date
Naproxen	Aleve®	Bayer	Aug 2018	https://www.fachinfo.de/api/fachinfo/pdf/005450	11 Aug 2021
Nebivolol	Nebivolol PUREN	Puren	Oct 2020	https://www.gelbe-liste.de/produkte/Nebivolol-PUREN-5-mg-Tabletten_1185511/fachinformation	23 Aug 2021
Nitrofurantoin	Nitrofurantoin-ratiopharm®	Ratiopharm	Jan 2019	https://www.fachinfo.de/api/fachinfo/pdf/001489	11 Aug 2021
Olanzapine	Zyprexa	Lilly	Oct 2020	https://www.fachinfo.de/api/fachinfo/pdf/000024	12 Aug 2021
Omeprazole	Omeprazol Hennig®	Hennig	Oct 2020	https://www.fachinfo.de/api/fachinfo/pdf/010849	11 Aug 2021
Ondansetron	Zofran®	Novartis Pharma	Nov 2020	https://www.fachinfo.de/api/fachinfo/pdf/020648	11 Aug 2021
Opicapone	Ongentys	Bial	Feb 2021	https://www.fachinfo.de/api/fachinfo/pdf/021261	12 Aug 2021
Oseltamivir	Tamiflu®	Roche	Apr 2020	https://www.fachinfo.de/api/fachinfo/pdf/014057	11 Aug 2021
Osilodrostat	Isturisa	Recordati Rare Diseases	May 2020	https://www.fachinfo.de/api/fachinfo/pdf/023059	12 Aug 2021
Oxycodone	Oxygesic®	Mundipharma	Nov 2020	https://www.fachinfo.de/api/fachinfo/pdf/010492	11 Aug 2021
Ozanimod	Zeposia®	Bristol Myers Squibb	Oct 2020	https://www.fachinfo.de/api/fachinfo/pdf/023003	12 Aug 2021
Pantoprazole	Pantozol®	Takeda	May 2021	https://www.fachinfo.de/api/fachinfo/pdf/006633	11 Aug 2021
Paracetamol	ben-u-ron	Bene	Mar 2021	https://www.fachinfo.de/api/fachinfo/pdf/005382	11 Aug 2021
Paroxetine	Seroxat	GlaxoSmithKline	Jan 2021	https://www.fachinfo.de/api/fachinfo/pdf/004747	11 Aug 2021
Pemigatinib	Pemazyre®	Incyte	Mar 2021	https://www.fachinfo.de/api/fachinfo/pdf/023344	12 Aug 2021
Perindopril	Coversum®	Servier Deutschland	Jun 2021	https://www.fachinfo.de/api/fachinfo/pdf/014949	11 Aug 2021
Pravastatin	Pravastatin-CT	AbZ Pharma	Apr 2021	https://www.fachinfo.de/api/fachinfo/pdf/014231	11 Aug 2021
Prednisolone	Decortin® H	Merck	Apr 2021	https://www.fachinfo.de/api/fachinfo/pdf/000525	12 Aug 2021
Pregabalin	Lyrica®	Pfizer	Mar 2021	https://www.fachinfo.de/api/fachinfo/pdf/008546	12 Aug 2021
Propranolol	Dociton®	Mibe	Aug 2019	https://www.fachinfo.de/api/fachinfo/pdf/007669	12 Aug 2021
Quetiapine	Seroquel®	AstraZeneca	Jun 2021	https://www.fachinfo.de/api/fachinfo/pdf/001458	12 Aug 2021
Quinine	Limptar®	Cassella-med	Sep 2019	https://www.fachinfo.de/api/fachinfo/pdf/003882	12 Aug 2021
Ramipril	Delix®	Sanofi	Apr 2021	https://www.fachinfo.de/api/fachinfo/pdf/005496	12 Aug 2021
Ranitidine	Ranitidin-ratiopharm®	Ratiopharm	Apr 2019	https://www.fachinfo.de/api/fachinfo/pdf/007176	12 Aug 2021
Remdesivir	Veklury®	Gilead	Jun 2021	https://www.fachinfo.de/api/fachinfo/pdf/023094	12 Aug 2021
Risdiplam	Evrysdi®	Roche	Mar 2021	https://www.fachinfo.de/api/fachinfo/pdf/023335	12 Aug 2021

Drug(s)	SmPC	Manufacturer	SmPC update	URL	Access date
Rivaroxaban	Xarelto®	Bayer	Jul 2021	https://www.fachinfo.de/api/fachinfo/pdf/013452	12 Aug 2021
Rosuvastatin	Crestor®	AstraZeneca	Jul 2021	https://www.fachinfo.de/api/fachinfo/pdf/011530	12 Aug 2021
Salbutamol	Ventilastin® Novolizer®	MEDA Pharma	Apr 2015	https://www.fachinfo.de/api/fachinfo/pdf/001246	12 Aug 2021
Selpercatinib	Retsevmo®	Lilly	Jun 2021	https://www.fachinfo.de/api/fachinfo/pdf/023262	12 Aug 2021
Sertraline	Zoloft®	Pfizer	Feb 2021	https://www.fachinfo.de/api/fachinfo/pdf/000405	12 Aug 2021
Sildenafil	Viagra®	Pfizer	Nov 2020	https://www.fachinfo.de/api/fachinfo/pdf/003927	12 Aug 2021
Simvastatin	Zocor®	Organon	Feb 2021	https://www.fachinfo.de/api/fachinfo/pdf/004748	12 Aug 2021
Sitagliptin	Januvia®	MSD	May 2020	https://www.fachinfo.de/api/fachinfo/pdf/010175	12 Aug 2021
Solifenacin	VesikurTM	Astellas	Dec 2018	https://www.fachinfo.de/api/fachinfo/pdf/008589	12 Aug 2021
Spironolactone	Aldactone®	Riemser	Jul 2018	https://www.fachinfo.de/api/fachinfo/pdf/012936	12 Aug 2021
Tamsulosin	Prostacure®	Mibe	Sep 2020	https://www.fachinfo.de/api/fachinfo/pdf/010214	12 Aug 2021
Telmisartan	Micardis®	Boehringer Ingelheim	Nov 2017	https://www.fachinfo.de/api/fachinfo/pdf/007242	12 Aug 2021
Thiamine	Vitamin B_1-ratiopharm®	Ratiopharm	Sep 2020	https://www.fachinfo.de/api/fachinfo/pdf/002289	12 Aug 2021
Timolol	Tim®-Ophtal®	Dr. Winzer	Apr 2021	https://www.fachinfo.de/api/fachinfo/pdf/002073	12 Aug 2021
Tiotropium bromide	Spiriva®	Boehringer Ingelheim	Oct 2018	https://www.fachinfo.de/api/fachinfo/pdf/006487	12 Aug 2021
Tivozanib	Fotivda	EUSA Pharm	Jun 2021	https://www.fachinfo.de/api/fachinfo/pdf/021832	25 Aug 2021
Topiramate	Topamax®	Janssen	Dec 2020	https://www.fachinfo.de/api/fachinfo/pdf/014374	12 Aug 2021
Torasemide	Unat	MEDA Pharma	Sep 2020	https://www.fachinfo.de/api/fachinfo/pdf/005096	12 Aug 2021
Tramadol	Tramal®	Grünenthal	Sep 2019	https://www.fachinfo.de/api/fachinfo/pdf/011112	12 Aug 2021
Trazodone	Trazodon-neuraxpharm®	neuraxpharm	Feb 2015	https://www.fachinfo.de/api/fachinfo/pdf/021416	12 Aug 2021
Trimethoprim/sulfamethoxazole	Eusaprim®	Aspen	Apr 2021	https://www.fachinfo.de/api/fachinfo/pdf/007468	12 Aug 2021
Trospium chloride	Spasmex®	Pfleger	Mar 2021	https://www.fachinfo.de/api/fachinfo/pdf/014933	12 Aug 2021
Valproic acid	Orfiril®	Desitin	Apr 2021	https://www.fachinfo.de/api/fachinfo/pdf/014608	12 Aug 2021
Valsartan	Diovan®	Novartis Pharma	Jun 2020	https://www.fachinfo.de/api/fachinfo/pdf/011820	12 Aug 2021
Valsartan/hydrochlorothiazide	CoDiovan®	Novartis Pharma	Aug 2020	https://www.fachinfo.de/api/fachinfo/pdf/005131	12 Aug 2021
Venlafaxine	Trevilor®	Pfizer	Mar 2021	https://www.fachinfo.de/api/fachinfo/pdf/004226	12 Aug 2021
Warfarin	Marcumar®	MEDA Pharma	Jun 2018	https://www.fachinfo.de/api/fachinfo/pdf/003056	12 Aug 2021

Drug(s)	SmPC	Manufacturer	SmPC update	URL	Access date
Zolpidem	Stilnox®	Sanofi	Aug 2020	https://www.fachinfo.de/api/fachinfo/pdf/004911	12 Aug 2021
