# Supplementary material for: Comprehensibility of Contraindications in German, UK and US Summaries of Product Characteristics/Prescribing Information—A Comparative Qualitative and Quantitative Analysis
Source: J Clin Med. 2022 Jul 18;11(14):4167. doi: 10.3390/jcm11144167 (PMC9316253; doi:10.3390/jcm11144167)
Supplement: Supplementary file 1 [file jcm-11-04167-s001.zip › Supplemental Table S2_20220310.pdf]

Supplemental Table S2. List of 158 UK Summaries of Product Characteristics (SmPCs) included in the analysis

Drug(s)	SmPC	Manufacturer	SmPC update	URL	Access date
Alendronic acid	Fosamax	Organon Pharma	Sep 2020	https://www.medicines.org.uk/emc/product/1281/smpc	23 Aug 2021
Allopurinol	Zyloric	Aspen	Feb 2019	https://www.medicines.org.uk/emc/product/7487/smpc	23 Aug 2021
Alprazolam	Xanax	Upjohn	Aug 2021	https://www.medicines.org.uk/emc/product/1656/smpc	23 Aug 2021
Amisulpride	Solian	Sanofi	Mar 2021	https://www.medicines.org.uk/emc/product/4091/smpc	23 Aug 2021
Amitriptyline	[generic]	Flamingo Pharma	May 2020	https://www.medicines.org.uk/emc/product/12612/smpc	23 Aug 2021
Amlodipine	ISTIN	Upjohn	Sep 2020	https://www.medicines.org.uk/emc/product/2925/smpc	23 Aug 2021
Amoxicillin	[generic]	Flamingo Pharma	Apr 2021	https://www.medicines.org.uk/emc/product/11312/smpc	23 Aug 2021
Amoxicillin/clavulanic acid	Augmentin	GlaxoSmithKline	May 2021	https://www.medicines.org.uk/emc/product/281/smpc	23 Aug 2021
Apixaban	Eliquis	Bristol Myers Squibb / Pfizer	May 2021	https://www.medicines.org.uk/emc/product/2878/smpc	23 Aug 2021
Aspirin	Aspirin	Accord	Jul 2021	https://www.medicines.org.uk/emc/product/11864/smpc	23 Aug 2021
Atenolol	Tenormin	AstraZeneca	Jun 2021	https://www.medicines.org.uk/emc/product/3861/smpc	23 Aug 2021
Atorvastatin	Lipitor	Upjohn	Jul 2020	https://www.medicines.org.uk/emc/product/5239/smpc	23 Aug 2021
Azithromycin	Zithromax	Pfizer	Dec 2020	https://www.medicines.org.uk/emc/product/1073/smpc	23 Aug 2021
Beclometasone	Qvar	Teva	Nov 2019	https://www.medicines.org.uk/emc/product/582/smpc	23 Aug 2021
Belantamab mafodotin	Blenrep	GlaxoSmithKline	Aug 2021	https://www.medicines.org.uk/emc/product/12545	23 Aug 2021
Bempedoic acid	Nilemdo	Daiichi Sankyo	Apr 2020	https://www.medicines.org.uk/emc/product/11743/smpc	23 Aug 2021
Betamethasone	Audavate	Accord	Feb 2020	https://www.medicines.org.uk/emc/product/5023/smpc	23 Aug 2021
Bisoprolol	Cardicor	Merck	Nov 2020	https://www.medicines.org.uk/emc/product/7763/smpc	23 Aug 2021
Botulinum toxin type A	Botox	Allergan	Dec 2020	https://www.medicines.org.uk/emc/product/436/smpc	23 Aug 2021
Brolucizumab	Beovu	Novartis Pharmaceuticals	Oct 2020	https://www.medicines.org.uk/emc/product/11145	23 Aug 2021
Budesonide	Pulmicort Turbohaler	AstraZeneca	Jun 2017	https://www.medicines.org.uk/emc/product/1386/smpc	23 Aug 2021
Bupropion	Zyban	GlaxoSmithKline	Jun 2021	https://www.medicines.org.uk/emc/product/3827/smpc	23 Aug 2021
Buspirone	[generic]	Accord	May 2021	https://www.medicines.org.uk/emc/product/5735/smpc	23 Aug 2021
Candesartan	Amias	Takeda	Jul 2020	https://www.medicines.org.uk/emc/product/11495/smpc	23 Aug 2021
Carvedilol	[generic]	Mylan	Mar 2017	https://www.medicines.org.uk/emc/product/2547/smpc	23 Aug 2021

Drug(s)	SmPC	Manufacturer	SmPC update	URL	Access date
Cefalexin	[generic]	Flynn Pharma	Feb 2021	https://www.medicines.org.uk/emc/product/9141/smpc	23 Aug 2021
Cefiderocol	Fetcroja	Shionogi	Apr 2020	https://www.medicines.org.uk/emc/product/11771	23 Aug 2021
Chlortalidone	Hylaton	Morningside Healthcare	Nov 2020	https://www.medicines.org.uk/emc/product/11982/smpc	23 Aug 2021
Citalopram	Cipramil	Lundbeck	Jan 2021	https://www.medicines.org.uk/emc/product/992/smpc	23 Aug 2021
Clonazepam	Clonazepam Rosemont	Rosemont Pharmaceuticals	Apr 2020	https://www.medicines.org.uk/emc/product/6022/smpc	23 Aug 2021
Clonidine	[generic]	Thame Laboratories	Jul 2020	https://www.medicines.org.uk/emc/product/9616/smpc	23 Aug 2021
Clopidogrel	Plavix	Sanofi	Jan 2021	https://www.medicines.org.uk/emc/product/5934/smpc	23 Aug 2021
Codeine phosphate/paracetamol	Co-codamol	Zentiva	Apr 2021	https://www.medicines.org.uk/emc/product/12502/smpc	23 Aug 2021
Cyanocobalamin	Orobalin	Northumbria Pharma	Apr 2021	https://www.medicines.org.uk/emc/product/11887/smpc	23 Aug 2021
Dexamethasone	[generic]	Aspen	Nov 2018	https://www.medicines.org.uk/emc/product/5370/smpc	23 Aug 2021
Diazepam	Stesolid	Accord	Nov 2019	https://www.medicines.org.uk/emc/product/104/smpc	23 Aug 2021
Diclofenac	Voltarol	Novartis Pharmaceuticals	Nov 2020	https://www.medicines.org.uk/emc/product/4333/smpc	23 Aug 2021
Digoxin	Lanoxin	Aspen	Feb 2020	https://www.medicines.org.uk/emc/product/5461/smpc	23 Aug 2021
Diltiazem	Slozem	Zentiva	Jul 2021	https://www.medicines.org.uk/emc/product/11705/smpc	23 Aug 2021
Dostarlimab	Jemperli	GlaxoSmithKline	Jul 2021	https://www.medicines.org.uk/emc/product/12669	23 Aug 2021
Doxazosin	Cardura	Upjohn	Jan 2021	https://www.medicines.org.uk/emc/product/6956/smpc	23 Aug 2021
Doxycycline	Vibramycin-D	Pfizer	Jul 2021	https://www.medicines.org.uk/emc/product/6262/smpc	23 Aug 2021
Duloxetine	Cymbalta	Eli Lilly and Company	Jun 2020	https://www.medicines.org.uk/emc/product/7450/smpc	23 Aug 2021
Edoxaban	Lixiana	Daiichi Sankyo	Nov 2020	https://www.medicines.org.uk/emc/product/6905/smpc	23 Aug 2021
Elexacaftor/ivacaftor/tezacaftor	Kaftrio	Vertex Pharmaceuticals	May 2021	https://www.medicines.org.uk/emc/product/11724	23 Aug 2021
Empagliflozin	Jardiance	Boehringer Ingelheim	Jul 2021	https://www.medicines.org.uk/emc/product/7703/smpc	23 Aug 2021
Enalapril	Innovace	Organon Pharma	Jun 2021	https://www.medicines.org.uk/emc/product/10544/smpc	23 Aug 2021
Enoxaparin sodium	Clexane	Sanofi	May 2020	https://www.medicines.org.uk/emc/product/1695/smpc	23 Aug 2021
Escitalopram	Cipralex	Lundbeck	Jun 2020	https://www.medicines.org.uk/emc/product/7716/smpc	23 Aug 2021
Esomeprazole	Nexium	AstraZeneca	Jan 2021	https://www.medicines.org.uk/emc/product/4658/smpc	23 Aug 2021
Estradiol	Elleste	Mylan	Sep 2020	https://www.medicines.org.uk/emc/product/9513/smpc	23 Aug 2021
Ethinylestradiol/norethisterone	Synphase	Pfizer	Jul 2021	https://www.medicines.org.uk/emc/product/1579/smpc	23 Aug 2021

Drug(s)	SmPC	Manufacturer	SmPC update	URL	Access date
Ethinylestradiol/norgestimate	Lizinna	Morningside Healthcare	Jul 2019	https://www.medicines.org.uk/emc/product/9573	23 Aug 2021
Ezetimibe	Ezetrol	Organon Pharma	Aug 2020	https://www.medicines.org.uk/emc/product/6792/smpc	23 Aug 2021
Felodipine	Plendil	AstraZeneca	May 2021	https://www.medicines.org.uk/emc/product/7544/smpc	23 Aug 2021
Fenofibrate	Supralip	Mylan	Aug 2020	https://www.medicines.org.uk/emc/product/2198/smpc	23 Aug 2021
Fexofenadine	Telfast	Sanofi	Oct 2019	https://www.medicines.org.uk/emc/product/6431/smpc	23 Aug 2021
Finasteride	Proscar	Organon Pharma	Apr 2021	https://www.medicines.org.uk/emc/product/1008/smpc	23 Aug 2021
Fluoxetine	[generic]	Flamingo Pharma	Jan 2021	https://www.medicines.org.uk/emc/product/10625/smpc	23 Aug 2021
Fluticasone	Avamys	GlaxoSmithKline	Jan 2021	https://www.medicines.org.uk/emc/product/6439/smpc	23 Aug 2021
Fluticasone/salmeterol	Stalpex	Glenmark Pharmaceuticals Europe	Apr 2021	https://www.medicines.org.uk/emc/product/9682/smpc	23 Aug 2021
Folic acid	[generic]	Wockhardt	Mar 2018	https://www.medicines.org.uk/emc/product/588/smpc	23 Aug 2021
Formoterol	Foradil	Novartis Pharmaceuticals	Dec 2019	https://www.medicines.org.uk/emc/product/1030/smpc	23 Aug 2021
Formoterol/budesonide	Symbicort Turbohaler	AstraZeneca	Feb 2021	https://www.medicines.org.uk/emc/product/1327/smpc	23 Aug 2021
Furosemide	[generic]	Ipca Laboratories	May 2020	https://www.medicines.org.uk/emc/product/12129/smpc	23 Aug 2021
Gabapentin	Neurontin	Upjohn	Feb 2021	https://www.medicines.org.uk/emc/product/3198/smpc	23 Aug 2021
Glimepiride	[generic]	Brown & Burk	Dec 2020	https://www.medicines.org.uk/emc/product/10745/smpc	23 Aug 2021
Hydrocortisone	[generic]	Accord	Jul 2021	https://www.medicines.org.uk/emc/product/5036/smpc	23 Aug 2021
Hydroxocobalamin	[generic]	G.L. Pharma / Kent Pharma	Jul 2018	https://www.medicines.org.uk/emc/product/12678/smpc	23 Aug 2021
Ibuprofen	Anadin	GlaxoSmithKline	Mar 2021	https://www.medicines.org.uk/emc/product/3876/smpc	23 Aug 2021
Indapamide	Natrilix	Servier Laboratories	Apr 2021	https://www.medicines.org.uk/emc/product/1151/smpc	23 Aug 2021
Insulin aspart	NovoMix	Novo Nordisk	Sep 2020	https://www.medicines.org.uk/emc/product/1600/smpc	23 Aug 2021
Insulin glargine	Lantus	Sanofi	Jan 2021	https://www.medicines.org.uk/emc/product/8098/smpc	23 Aug 2021
Insulin human	Insuman	Sanofi	Jan 2021	https://www.medicines.org.uk/emc/product/4104/smpc	23 Aug 2021
Insulin lispro	Humalog	Eli Lilly and Company	Sep 2020	https://www.medicines.org.uk/emc/product/7473/smpc	23 Aug 2021
Irbesartan	Aprovel	Sanofi	Jul 2021	https://www.medicines.org.uk/emc/product/3052/smpc	23 Aug 2021
Isatuximab	Sarclisa	Sanofi Genzyme	Aug 2021	https://www.medicines.org.uk/emc/product/11535	23 Aug 2021
Isosorbide mononitrate	Isodur	Galen	Jun 2020	https://www.medicines.org.uk/emc/product/11062/smpc	23 Aug 2021
Lactulose	Duphalac	Mylan	Aug 2020	https://www.medicines.org.uk/emc/product/5525/smpc	23 Aug 2021

Drug(s)	SmPC	Manufacturer	SmPC update	URL	Access date
Lamotrigine	Lamictal	GlaxoSmithKline	Feb 2021	https://www.medicines.org.uk/emc/product/1286/smpc	23 Aug 2021
Lansoprazole	Zoton	Pfizer	Mar 2021	https://www.medicines.org.uk/emc/product/1969/smpc	23 Aug 2021
Latanoprost	Xalatan	Upjohn	Dec 2020	https://www.medicines.org.uk/emc/product/1616	23 Aug 2021
Levetiracetam	Keppra	UCB Pharma	Jan 2021	https://www.medicines.org.uk/emc/product/2291/smpc	23 Aug 2021
Levonogestrel/ethinylestradiol	Levest	Morningside Healthcare	Jun 2021	https://www.medicines.org.uk/emc/product/9565/smpc	23 Aug 2021
Levothyroxine sodium	[generic]	Glenmark Pharmaceuticals Europe	Apr 2021	https://www.medicines.org.uk/emc/product/12781/smpc	23 Aug 2021
Lisdexamfetamine	Elvanse	Takeda	Jan 2021	https://www.medicines.org.uk/emc/product/7498/smpc	23 Aug 2021
Lisinopril	Zestril	AstraZeneca	Aug 2019	https://www.medicines.org.uk/emc/product/7610/smpc	23 Aug 2021
Lisinopril/hydrochlorothiazide	Zestoretic	AstraZeneca	Jul 2020	https://www.medicines.org.uk/emc/product/5502/smpc	23 Aug 2021
Loratadine	Clarityn	Bayer plc	Aug 2018	https://www.medicines.org.uk/emc/product/3506/smpc	23 Aug 2021
Lorazepam	Ativan	Pfizer	Sep 2020	https://www.medicines.org.uk/emc/product/5473/smpc	23 Aug 2021
Losartan	Cozaar	Organon Pharma	Dec 2018	https://www.medicines.org.uk/emc/product/7798/smpc	23 Aug 2021
Losartan/hydrochlorothiazide	Cozaar comp	Organon Pharma	Oct 2020	https://www.medicines.org.uk/emc/product/7781/smpc	23 Aug 2021
Macrogol	Movicol	Norgine	Jun 2021	https://www.medicines.org.uk/emc/product/7366/smpc	23 Aug 2021
Meloxicam	[generic]	Fontus Health	Jul 2017	https://www.medicines.org.uk/emc/product/8574/smpc	23 Aug 2021
Mesalazine	Pentasa	Ferring Pharmaceuticals	May 2021	https://www.medicines.org.uk/emc/product/4778/smpc	23 Aug 2021
Metformin	Glucophage	Merck	Aug 2019	https://www.medicines.org.uk/emc/product/7759/smpc	23 Aug 2021
Metformin/sitagliptin	Janumet	MSD	Jan 2021	https://www.medicines.org.uk/emc/product/564/smpc	23 Aug 2021
Methotrexate	[generic]	Orion Pharma	Mar 2021	https://www.medicines.org.uk/emc/product/10985/smpc	23 Aug 2021
Methylphenidate	Ritalin	Novartis Pharmaceuticals	Nov 2020	https://www.medicines.org.uk/emc/product/11098/smpc	23 Aug 2021
Metoprolol	[generic]	Aurobindo Pharma - Milpharm	Nov 2020	https://www.medicines.org.uk/emc/product/5199/smpc	23 Aug 2021
Mirtazapine	[generic]	Rosemont Pharmaceuticals	Jul 2021	https://www.medicines.org.uk/emc/product/2023/smpc	23 Aug 2021
Mometasone	Elocon	Organon Pharma	Mar 2021	https://www.medicines.org.uk/emc/product/1139/smpc	23 Aug 2021
Montelukast	Singulair	Organon Pharma	Mar 2020	https://www.medicines.org.uk/emc/product/198/smpc	23 Aug 2021
Morphine	Zomorph	Ethypharm	May 2021	https://www.medicines.org.uk/emc/product/1420/smpc	23 Aug 2021
Naproxen	[generic]	Aurobindo Pharma - Milpharm	Oct 2020	https://www.medicines.org.uk/emc/product/543/smpc	23 Aug 2021
Nebivolol	[generic]	Accord	May 2021	https://www.medicines.org.uk/emc/product/5828/smpc	23 Aug 2021

Drug(s)	SmPC	Manufacturer	SmPC update	URL	Access date
Nitrofurantoin	Furadantin	Advanz Pharma	Mar 2019	https://www.medicines.org.uk/emc/product/5749/smpc	23 Aug 2021
Olanzapine	[generic]	Accord	Jun 2021	https://www.medicines.org.uk/emc/product/4769/smpc	23 Aug 2021
Omeprazole	Pyrocalm	Dexcel Pharma	Jul 2021	https://www.medicines.org.uk/emc/product/9154/smpc	23 Aug 2021
Ondansetron	Zofran	Novartis Pharmaceuticals	Sep 2020	https://www.medicines.org.uk/emc/product/11167/smpc	23 Aug 2021
Opicapone	Ongentys	Bial Pharma	Feb 2021	https://www.medicines.org.uk/emc/product/7386	23 Aug 2021
Oseltamivir	Tamiflu	Roche Products	Jan 2021	https://www.medicines.org.uk/emc/product/1194/smpc	23 Aug 2021
Osilodrostat	Isturia	Recordati Rare Diseases	May 2020	https://www.medicines.org.uk/emc/product/11588/smpc	23 Aug 2021
Oxycodone	Lynlor	Accord	Aug 2021	https://www.medicines.org.uk/emc/product/1196/smpc	23 Aug 2021
Ozanimod	Zeposia	Bristol-Myers Squibb Pharmaceuticals	Jan 2021	https://www.medicines.org.uk/emc/product/11908/smpc	23 Aug 2021
Pantoprazole	Protium	Takeda	Apr 2020	https://www.medicines.org.uk/emc/product/2241/smpc	23 Aug 2021
Paracetamol	Panadol	GlaxoSmithKline Consumer Healthcare	Jul 2021	https://www.medicines.org.uk/emc/product/6482/smpc	23 Aug 2021
Paroxetine	[generic]	Aurobindo Pharma - Milpharm	Jun 2021	https://www.medicines.org.uk/emc/product/539/smpc	23 Aug 2021
Pemigatinib	Pemazyre	Incyte Biosciences	Apr 2021	https://www.medicines.org.uk/emc/product/12485	23 Aug 2021
Perindopril	[generic]	Sandoz	Nov 2020	https://www.medicines.org.uk/emc/product/12742/smpc	23 Aug 2021
Pravastatin	[generic]	Aurobindo Pharma - Milpharm	Jun 2018	https://www.medicines.org.uk/emc/product/5322/smpc	23 Aug 2021
Prednisolone	[generic]	Accord	Jul 2021	https://www.medicines.org.uk/emc/product/5885/smpc	23 Aug 2021
Pregabalin	Lyrica	Upjohn	Mar 2021	https://www.medicines.org.uk/emc/product/10310/smpc	23 Aug 2021
Propranolol	[generic]	Thame Laboratories	Jul 2020	https://www.medicines.org.uk/emc/product/9857/smpc	23 Aug 2021
Quetiapine	Seroquel	Luye Pharma	Sep 2020	https://www.medicines.org.uk/emc/product/7547/smpc	23 Aug 2021
Quinine	[generic]	Accord	Oct 2019	https://www.medicines.org.uk/emc/product/4554/smpc	23 Aug 2021
Ramipril	Tritace	Sanofi	Jun 2021	https://www.medicines.org.uk/emc/product/2757/smpc	23 Aug 2021
Ranitidine	Zantac	Omega Pharma	Sep 2019	https://www.medicines.org.uk/emc/product/6491/smpc	23 Aug 2021
Remdesivir	Veklury	Gilead Sciences	Jul 2021	https://www.medicines.org.uk/emc/product/11597	23 Aug 2021
Risdiplam	Evrysdi	Roche Products	May 2021	https://www.medicines.org.uk/emc/product/12582	23 Aug 2021
Rivaroxaban	Xarelto	Bayer plc	Jan 2021	https://www.medicines.org.uk/emc/product/2793/smpc	23 Aug 2021
Rosuvastatin	Crestor	AstraZeneca	Aug 2021	https://www.medicines.org.uk/emc/product/7554/smpc	23 Aug 2021
Salbutamol	Ventolin	GlaxoSmithKline	Nov 2020	https://www.medicines.org.uk/emc/product/852/smpc	23 Aug 2021

Drug(s)	SmPC	Manufacturer	SmPC update	URL	Access date
Selpercatinib	Retsevmo	Eli Lilly and Company	Feb 2021	https://www.medicines.org.uk/emc/product/12196/smpc	23 Aug 2021
Sertraline	Lustral	Upjohn	Mar 2021	https://www.medicines.org.uk/emc/product/2835/smpc	23 Aug 2021
Sildenafil	Viagra	Upjohn	Nov 2020	https://www.medicines.org.uk/emc/product/7978/smpc	23 Aug 2021
Simvastatin	Zocor	Organon Pharma	Jan 2021	https://www.medicines.org.uk/emc/product/7789/smpc	23 Aug 2021
Sitagliptin	Januvia	MSD	Jan 2021	https://www.medicines.org.uk/emc/product/7887/smpc	23 Aug 2021
Solifenacin	Vesicare	Astellas Pharma	Nov 2019	https://www.medicines.org.uk/emc/product/7493/smpc	23 Aug 2021
Spironolactone	Aldactone	Pfizer	May 2019	https://www.medicines.org.uk/emc/product/2898/smpc	23 Aug 2021
Tamsulosin	[generic]	Zentiva	Jul 2021	https://www.medicines.org.uk/emc/product/9245/smpc	23 Aug 2021
Telmisartan	Micardis	Boehringer Ingelheim	Nov 2020	https://www.medicines.org.uk/emc/product/3164/smpc	23 Aug 2021
Thiamine	[generic]	Flamingo Pharma	Mar 2019	https://www.medicines.org.uk/emc/product/11215/smpc	23 Aug 2021
Timolol	Eysano	Aspire Pharma	Jan 2020	https://www.medicines.org.uk/emc/product/2534/smpc	23 Aug 2021
Tiotropium bromide	Spiriva Respimat	Boehringer Ingelheim	Dec 2020	https://www.medicines.org.uk/emc/product/407/smpc	23 Aug 2021
Tivozanib	Fotivda	Eusa Pharm	Jun 2021	https://www.medicines.org.uk/emc/product/8996/smpc	23 Aug 2021
Topiramate	Topamax	Janssen-Cilag	Jan 2021	https://www.medicines.org.uk/emc/product/1976/smpc	23 Aug 2021
Torasemide	Torem	Mylan	Feb 2018	https://www.medicines.org.uk/emc/product/6663/smpc	23 Aug 2021
Tramadol	[generic]	Accord Healthcare	Jul 2021	https://www.medicines.org.uk/emc/product/6116/smpc	23 Aug 2021
Trazodone	Molipaxin	Zentiva	Mar 2021	https://www.medicines.org.uk/emc/product/4197/smpc	23 Aug 2021
Trimethoprim/sulfamethoxazole	Co-Trimoxazole	Accord	Aug 2021	https://www.medicines.org.uk/emc/product/5752/smpc	23 Aug 2021
Trospium chloride	Regurin	Mylan	Sep 2020	https://www.medicines.org.uk/emc/product/6644/smpc	23 Aug 2021
Valproic acid	Convulex	Gerot Lannach	Jan 2019	https://www.medicines.org.uk/emc/product/11236/smpc	23 Aug 2021
Valsartan	Diovan	Novartis Pharmaceuticals	Jul 2021	https://www.medicines.org.uk/emc/product/5991/smpc	23 Aug 2021
Valsartan/hydrochlorothiazide	Co-Diovan	Novartis Pharmaceuticals	Aug 2020	https://www.medicines.org.uk/emc/product/7744/smpc	23 Aug 2021
Venlafaxine	Effexor	Upjohn	Mar 2021	https://www.medicines.org.uk/emc/product/5059/smpc	23 Aug 2021
Warfarin	[generic]	Ranbaxy	Feb 2017	https://www.medicines.org.uk/emc/product/3064/smpc	23 Aug 2021
Zolpidem	[generic]	Zentiva	Sep 2020	https://www.medicines.org.uk/emc/product/3975/smpc	23 Aug 2021
