# Supplementary material for: Comprehensibility of Contraindications in German, UK and US Summaries of Product Characteristics/Prescribing Information—A Comparative Qualitative and Quantitative Analysis
Source: J Clin Med. 2022 Jul 18;11(14):4167. doi: 10.3390/jcm11144167 (PMC9316253; doi:10.3390/jcm11144167)
Supplement: Supplementary file 1 [file jcm-11-04167-s001.zip › Supplemental Table S3_20220310.pdf]

Supplemental Table S3. List of 158 US Prescribing Information (PI) included in the analysis

Drug(s)	PI	PI update	URL	Access date
Alendronic acid	Fosamax	Jun 2021	https://www.drugs.com/pro/fosamax.html	23 Aug 2021
Allopurinol	Zyloprim	Nov 2020	https://www.drugs.com/pro/zyloprim.html	23 Aug 2021
Alprazolam	Xanax	Mar 2021	https://www.drugs.com/pro/xanax.html	23 Aug 2021
Amisulpride	Barhemsys	Jun 2021	https://www.drugs.com/pro/barhemsys.html	23 Aug 2021
Amitriptyline	Elavil	Mar 2021	https://www.drugs.com/pro/elavil.html	23 Aug 2021
Amlodipine	Norvasc	Feb 2021	https://www.drugs.com/pro/norvasc.html	23 Aug 2021
Amoxicillin	Amoxil	Mar 2021	https://www.drugs.com/pro/amoxil.html	23 Aug 2021
Amoxicillin/clavulanic acid	Augmentin	Aug 2020	https://www.drugs.com/pro/augmentin.html	23 Aug 2021
Apixaban	Eliquis	Jun 2021	https://www.drugs.com/pro/eliquis.html	23 Aug 2021
Aspirin	Durlaza	Aug 2020	https://www.drugs.com/pro/durlaza.html	23 Aug 2021
Atenolol	Tenormin	Dec 2020	https://www.drugs.com/pro/tenormin.html	23 Aug 2021
Atorvastatin	Lipitor	Aug 2021	https://www.drugs.com/pro/lipitor.html	23 Aug 2021
Azithromycin	Zithromax	Apr 2020	https://www.drugs.com/pro/zithromax.html	23 Aug 2021
Beclometasone	Qvar	Jan 2021	https://www.drugs.com/pro/qvar.html	23 Aug 2021
Belantamab mafodotin	Blenrep	Aug 2020	https://www.drugs.com/pro/blenrep.html	23 Aug 2021
Bempedoic acid	Nexletol	Mar 2020	https://www.drugs.com/pro/nexletol.html	23 Aug 2021
Betamethasone	Celestone	Jul 2021	https://www.drugs.com/pro/celestone.html	23 Aug 2021
Bisoprolol	Zebeta	Apr 2021	https://www.drugs.com/pro/zebeta.html	23 Aug 2021
Botulinum toxin type A	Botox	Jul 2021	https://www.drugs.com/pro/botox.html	23 Aug 2021
Brolucizumab	Beovu	Feb 2021	https://www.drugs.com/pro/beovu-injection.html	23 Aug 2021
Budesonide	Pulmicort	Oct 2019	https://www.drugs.com/pro/pulmicort.html	23 Aug 2021
Bupropion	Wellbutrin	Mar 2021	https://www.drugs.com/pro/wellbutrin.html	23 Aug 2021
Buspirone	BuSpar	Oct 2020	https://www.drugs.com/pro/buspar.html	23 Aug 2021
Candesartan	Atacand	Jun 2020	https://www.drugs.com/pro/atacand.html	23 Aug 2021
Carvedilol	Coreg	Aug 2020	https://www.drugs.com/pro/coreg.html	23 Aug 2021
Cefalexin	Keflex	May 2021	https://www.drugs.com/pro/keflex.html	23 Aug 2021

Drug(s)	PI	PI update	URL	Access date
Cefiderocol	Fetroja	Sep 2020	https://www.drugs.com/pro/fetroja.html	23 Aug 2021
Chlortalidone	Chlorthalidone	Dec 2020	https://www.drugs.com/pro/chlorthalidone.html	23 Aug 2021
Citalopram	Celexa	Jul 2021	https://www.drugs.com/pro/celexa.html	23 Aug 2021
Clonazepam	Klonopin	Feb 2021	https://www.drugs.com/pro/klonopin.html	23 Aug 2021
Clonidine	Catapres	Jan 2020	https://www.drugs.com/pro/catapres.html	23 Aug 2021
Clopidogrel	Plavix	Mar 2021	https://www.drugs.com/pro/plavix.html	23 Aug 2021
Codeine phosphate/paracetamol	Tylenol With Codeine	Dec 2020	https://www.drugs.com/pro/tylenol-with-codeine.html	23 Aug 2021
Cyanocobalamin	Cyanocobalamin	Sep 2020	https://www.drugs.com/pro/cyanocobalamin.html	23 Aug 2021
Dexamethasone	Decadron	Jan 2021	https://www.drugs.com/pro/decadron.html	23 Aug 2021
Diazepam	Valium	Nov 2019	https://www.drugs.com/pro/valium.html	23 Aug 2021
Diclofenac	Voltaren	Jan 2021	https://www.drugs.com/pro/voltaren.html	23 Aug 2021
Digoxin	Lanoxin	Oct 2020	https://www.drugs.com/pro/lanoxin.html	23 Aug 2021
Diltiazem	Cardizem	Jun 2020	https://www.drugs.com/pro/cardizem.html	23 Aug 2021
Dostarlimab	Jemperli	Aug 2021	https://www.drugs.com/pro/jemperli.html	23 Aug 2021
Doxazosin	Cardura	Jul 2021	https://www.drugs.com/pro/cardura.html	23 Aug 2021
Doxycycline	Vibramycin	Jul 2021	https://www.drugs.com/pro/vibramycin.html	23 Aug 2021
Duloxetine	Cymbalta	Nov 2020	https://www.drugs.com/pro/cymbalta.html	23 Aug 2021
Edoxaban	Savaysa	Mar 2021	https://www.drugs.com/pro/savaysa.html	23 Aug 2021
Elexacaftor/vacaftor/tezacaftor	Trikafta	Jun 2021	https://www.drugs.com/pro/trikafta.html	23 Aug 2021
Empagliflozin	Jardiance	Feb 2021	https://www.drugs.com/pro/jardiance.html	23 Aug 2021
Enalapril	Vasotec	Dec 2020	https://www.drugs.com/pro/vasotec.html	23 Aug 2021
Enoxaparin sodium	Lovenox	Apr 2020	https://www.drugs.com/pro/lovenox.html	23 Aug 2021
Escitalopram	Lexapro	Aug 2020	https://www.drugs.com/pro/lexapro.html	23 Aug 2021
Esomeprazole	Nexium	Nov 2020	https://www.drugs.com/pro/nexium.html	23 Aug 2021
Estradiol	Delestrogen	Aug 2020	https://www.drugs.com/pro/delestrogen.html	23 Aug 2021
Ethinylestradiol/norethisterone	Loestrin	Nov 2020	https://www.drugs.com/pro/loestrin.html	23 Aug 2021
Ethinylestradiol/norgestimate	Norgestimate and Ethinyl Estradiol	Apr 2020	https://www.drugs.com/pro/norgestimate-and-ethinyl-estradiol.html	23 Aug 2021

Drug(s)	PI	PI update	URL	Access date
Ezetimibe	Zetia	Jun 2021	https://www.drugs.com/pro/zetia.html	23 Aug 2021
Felodipine	Plendil	Sep 2020	https://www.drugs.com/pro/plendil.html	23 Aug 2021
Fenofibrate	Tricor	Jun 2021	https://www.drugs.com/pro/tricor.html	23 Aug 2021
Fexofenadine	Allegra	Feb 2021	https://www.drugs.com/pro/allegra.html	23 Aug 2021
Finasteride	Proscar	Jun 2021	https://www.drugs.com/pro/proscar.html	23 Aug 2021
Fluoxetine	Prozac Weekly	Sep 2019	https://www.drugs.com/pro/prozac-weekly.html	23 Aug 2021
Fluticasone	Flovent	Feb 2021	https://www.drugs.com/pro/flovent.html	23 Aug 2021
Fluticasone/salmeterol	Advair Diskus	Nov 2020	https://www.drugs.com/pro/advair-diskus.html	23 Aug 2021
Folic acid	Folic Acid	Feb 2021	https://www.drugs.com/pro/folic-acid.html	23 Aug 2021
Formoterol	Foradil	Feb 2021	https://www.drugs.com/pro/foradil.html	23 Aug 2021
Formoterol/budesonide	Symbicort	Jun 2021	https://www.drugs.com/pro/symbicort.html	23 Aug 2021
Furosemide	Lasix	Jan 2021	https://www.drugs.com/pro/lasix.html	23 Aug 2021
Gabapentin	Neurontin	Dec 2020	https://www.drugs.com/pro/neurontin.html	23 Aug 2021
Glimepiride	Amaryl	Jan 2021	https://www.drugs.com/pro/amaryl.html	23 Aug 2021
Hydrocortisone	Hydrocortisone	Mar 2021	https://www.drugs.com/pro/hydrocortisone.html	23 Aug 2021
Hydroxocobalamin	Hydroxocobalamin	Nov 2020	https://www.drugs.com/pro/hydroxocobalamin.html	23 Aug 2021
Ibuprofen	Ibuprofen	Sep 2020	https://www.drugs.com/pro/ibuprofen.html	23 Aug 2021
Indapamide	Lozol	Aug 2020	https://www.drugs.com/pro/lozol.html	23 Aug 2021
Insulin aspart	Novolog	Mar 2021	https://www.drugs.com/pro/novolog-injection.html	23 Aug 2021
Insulin glargine	Lantus	Jan 2021	https://www.drugs.com/pro/lantus.html	23 Aug 2021
Insulin human	Humulin R	Oct 2020	https://www.drugs.com/pro/humulin-r.html	23 Aug 2021
Insulin lispro	Humalog	May 2021	https://www.drugs.com/pro/humalog.html	23 Aug 2021
Irbesartan	Avapro	May 2021	https://www.drugs.com/pro/avapro.html	23 Aug 2021
Isatuximab	Sarclisa	Apr 2021	https://www.drugs.com/pro/sarclisa.html	23 Aug 2021
Isosorbide mononitrate	Isosorbide Mononitrate	Dec 2020	https://www.drugs.com/pro/isosorbide-mononitrate.html	23 Aug 2021
Lactulose	Lactulose	May 2021	https://www.drugs.com/pro/lactulose.html	23 Aug 2021
Lamotrigine	Lamictal	Mar 2021	https://www.drugs.com/pro/lamictal.html	23 Aug 2021

Drug(s)	PI	PI update	URL	Access date
Lansoprazole	Prevacid	May 2021	https://www.drugs.com/pro/prevacid.html	23 Aug 2021
Latanoprost	Xalatan	Apr 2021	https://www.drugs.com/pro/xalatan.html	23 Aug 2021
Levetiracetam	Keppra	Oct 2020	https://www.drugs.com/pro/keppra.html	23 Aug 2021
Levonogestrel/ethinylestradiol	Nordette	Oct 2020	https://www.drugs.com/pro/nordette.html	23 Aug 2021
Levothyroxine sodium	Unithroid	May 2021	https://www.drugs.com/pro/unithroid.html	23 Aug 2021
Lisdexamfetamine	Vyvanse	Aug 2021	https://www.drugs.com/pro/vyvanse.html	23 Aug 2021
Lisinopril	Prinivil	Oct 2020	https://www.drugs.com/pro/prinivil.html	23 Aug 2021
Lisinopril/hydrochlorothiazide	Prinzide	Nov 2020	https://www.drugs.com/pro/prinzide.html	23 Aug 2021
Loratadine	Claritin	Dec 2020	https://www.accessdata.fda.gov/drugsatfda_docs/label/2019/021165s022,021300s019,021312s020,021563s008lbl.pdf	14 Sep 2021
Lorazepam	Ativan	Apr 2021	https://www.drugs.com/pro/ativan.html	23 Aug 2021
Losartan	Cozaar	Jun 2021	https://www.drugs.com/pro/cozaar.html	23 Aug 2021
Losartan/hydrochlorothiazide	Hyzaar	Jun 2021	https://www.drugs.com/pro/hyzaar.html	23 Aug 2021
Macrogol	Polyethylene Glycol	Oct 2020	https://www.drugs.com/pro/polyethylene-glycol.html	23 Aug 2021
Meloxicam	Mobic	Aug 2020	https://www.drugs.com/pro/mobic.html	23 Aug 2021
Mesalazine	Rowasa	Jun 2021	https://www.drugs.com/pro/rowasa.html	23 Aug 2021
Metformin	Glucophage	Apr 2021	https://www.drugs.com/pro/glucophage.html	23 Aug 2021
Metformin/sitagliptin	Janumet	Jun 2021	https://www.drugs.com/pro/janumet.html	23 Aug 2021
Methotrexate	Methotrexate Sodium	May 2021	https://www.drugs.com/pro/methotrexate-sodium.html	23 Aug 2021
Methylphenidate	Ritalin	Jun 2021	https://www.drugs.com/pro/ritalin.html	23 Aug 2021
Metoprolol	Lopressor	Jan 2021	https://www.drugs.com/pro/lopressor.html	23 Aug 2021
Mirtazapine	Remeron	Jun 2021	https://www.drugs.com/pro/remeron.html	23 Aug 2021
Mometasone	Elocon	Nov 2020	https://www.drugs.com/pro/elocon.html	23 Aug 2021
Montelukast	Singulair	Jun 2021	https://www.drugs.com/pro/singulair.html	23 Aug 2021
Morphine	Duramorph	Jan 2020	https://www.drugs.com/pro/duramorph.html	23 Aug 2021
Naproxen	Naprosyn	Apr 2021	https://www.drugs.com/pro/naprosyn.html	23 Aug 2021
Nebivolol	Bystolic	May 2020	https://www.drugs.com/pro/bystolic.html	23 Aug 2021
Nitrofurantoin	Furadantin	May 2021	https://www.drugs.com/pro/furadantin.html	23 Aug 2021

Drug(s)	PI	PI update	URL	Access date
Olanzapine	Zyprexa	Apr 2020	https://www.drugs.com/pro/zyprexa.html	23 Aug 2021
Omeprazole	Prilosec	Nov 2020	https://www.drugs.com/pro/prilosec.html	23 Aug 2021
Ondansetron	Zofran	Sep 2020	https://www.drugs.com/pro/zofran.html	23 Aug 2021
Opicapone	Ongentys	Apr 2020	https://www.drugs.com/pro/ongentys.html	23 Aug 2021
Oseltamivir	Tamiflu	Jun 2021	https://www.drugs.com/pro/tamiflu.html	23 Aug 2021
Osilodrostat	Isturisa	Mar 2020	https://www.drugs.com/pro/isturisa.html	23 Aug 2021
Oxycodone	Roxicodone	Mar 2021	https://www.drugs.com/pro/roxicodone.html	23 Aug 2021
Ozanimod	Zeposia	Jun 2021	https://www.drugs.com/pro/zeposia.html	23 Aug 2021
Pantoprazole	Protonix	Apr 2021	https://www.drugs.com/pro/protonix.html	23 Aug 2021
Paracetamol	Ofirmev	Mar 2018	https://www.accessdata.fda.gov/drugsatfda_docs/label/2018/022450s011lbl.pdf	23 Aug 2021
Paroxetine	Paxil	Jun 2021	https://www.drugs.com/pro/paxil.html	23 Aug 2021
Pemigatinib	Pemazyre	Jun 2021	https://www.drugs.com/pro/pemazyre.html	23 Aug 2021
Perindopril	Aceon	Apr 2021	https://www.drugs.com/pro/aceon.html	23 Aug 2021
Pravastatin	Pravachol	Oct 2020	https://www.drugs.com/pro/pravachol.html	23 Aug 2021
Prednisolone	Prednisolone	Jun 2020	https://www.drugs.com/pro/prednisolone-odt.html	23 Aug 2021
Pregabalin	Lyrica	Jun 2020	https://www.drugs.com/pro/lyrica.html	23 Aug 2021
Propranolol	Inderal	Dec 2020	https://www.drugs.com/pro/inderal.html	23 Aug 2021
Quetiapine	Seroquel	Sep 2020	https://www.drugs.com/pro/seroquel.html	23 Aug 2021
Quinine	Qualaquin	Jul 2021	https://www.drugs.com/pro/qualaquin.html	23 Aug 2021
Ramipril	Altace	Jul 2021	https://www.drugs.com/pro/altace.html	23 Aug 2021
Ranitidine	Ranitidine	Jan 2021	https://www.drugs.com/pro/ranitidine.html	23 Aug 2021
Remdesivir	Veklury	Feb 2021	https://www.accessdata.fda.gov/drugsatfda_docs/label/2021/214787s005lbl.pdf	23 Aug 2021
Risdiplam	Evrysdi	May 2021	https://www.drugs.com/pro/evrysdi.html	23 Aug 2021
Rivaroxaban	Xarelto	Jul 2021	https://www.drugs.com/pro/xarelto.html	23 Aug 2021
Rosuvastatin	Crestor	Sep 2020	https://www.drugs.com/pro/crestor.html	23 Aug 2021
Salbutamol	Ventolin	Feb 2021	https://www.drugs.com/pro/ventolin.html	23 Aug 2021
Selpercatinib	Retevmo	Jan 2021	https://www.drugs.com/pro/retevmo.html	23 Aug 2021

Drug(s)	PI	PI update	URL	Access date
Sertraline	Zoloft	Jul 2021	https://www.drugs.com/pro/zoloft.html	23 Aug 2021
Sildenafil	Viagra	Jul 2021	https://www.drugs.com/pro/viagra.html	23 Aug 2021
Simvastatin	Zocor	Dec 2020	https://www.drugs.com/pro/zocor.html	23 Aug 2021
Sitagliptin	Januvia	Oct 2020	https://www.drugs.com/pro/januvia.html	23 Aug 2021
Solifenacin	Vesicare	Jun 2020	https://www.drugs.com/pro/vesicare.html	23 Aug 2021
Spironolactone	Aldactone	Feb 2021	https://www.drugs.com/pro/aldactone.html	23 Aug 2021
Tamsulosin	Flomax	Dec 2019	https://www.drugs.com/pro/flomax.html	23 Aug 2021
Telmisartan	Micardis	Sep 2020	https://www.drugs.com/pro/micardis.html	23 Aug 2021
Thiamine	Thiamine Hydrochloride	Jun 2021	https://www.drugs.com/pro/thiamine-hydrochloride-injection.html	23 Aug 2021
Timolol	Blocadren	Jan 2021	https://www.drugs.com/pro/blocadren.html	23 Aug 2021
Tiotropium bromide	Spiriva	Oct 2019	https://www.drugs.com/pro/spiriva.html	23 Aug 2021
Tivozanib	Fotivda	Mar 2021	https://www.drugs.com/pro/fotivda.html	23 Aug 2021
Topiramate	Topamax	Jul 2021	https://www.drugs.com/pro/topamax.html	23 Aug 2021
Torasemide	Demadex	Jan 2021	https://www.drugs.com/pro/demadex.html	23 Aug 2021
Tramadol	Ultram	Mar 2021	https://www.drugs.com/pro/ultram.html	23 Aug 2021
Trazodone	Desyrel	Dec 2020	https://www.drugs.com/pro/desyrel.html	23 Aug 2021
Trimethoprim/sulfamethoxazole	Bactrim	Apr 2021	https://www.drugs.com/pro/bactrim.html	23 Aug 2021
Trospium chloride	Sanctura	Jun 2021	https://www.drugs.com/pro/sanctura.html	23 Aug 2021
Valproic acid	Depakene	Sep 2019	https://www.drugs.com/pro/depakene.html	23 Aug 2021
Valsartan	Diovan	Apr 2021	https://www.drugs.com/pro/diovan.html	23 Aug 2021
Valsartan/hydrochlorothiazide	Diovan HCT	Jun 2021	https://www.drugs.com/pro/diovan-hct.html	23 Aug 2021
Venlafaxine	Effexor	Dec 2020	https://www.drugs.com/pro/effexor.html	23 Aug 2021
Warfarin	Coumadin	Aug 2020	https://www.drugs.com/pro/coumadin.html	23 Aug 2021
Zolpidem	Ambien	Jul 2020	https://www.drugs.com/pro/ambien.html	23 Aug 2021
