# Supplementary material for: Comprehensibility of Contraindications in German, UK and US Summaries of Product Characteristics/Prescribing Information—A Comparative Qualitative and Quantitative Analysis
Source: J Clin Med. 2022 Jul 18;11(14):4167. doi: 10.3390/jcm11144167 (PMC9316253; doi:10.3390/jcm11144167)
Supplement: Supplementary file 1 [file jcm-11-04167-s001.zip › Supplemental Table S4_20220408.pdf]

Supplemental Table S4. Extraction and evaluation of CIs for example rivaroxaban (Xarelto) UK SmPC

SmPC (original text)	Individual absolute CIs (extraction)	Clarity	Codability
Hypersensitivity to the active substance or to any of the excipients listed in section 6.1.	hypersensitivity to the active substance	clear	not codable
	hypersensitivity to any of the excipients	clear	not codable
Active clinically significant bleeding. Lesion or condition, if considered to be a significant risk for major bleeding. This may include current or recent gastrointestinal ulceration, presence of malignant neoplasms at high risk of bleeding, recent brain or spinal injury, recentbrain, spinal or ophthalmic surgery, recent intracranial haemorrhage, known or suspected oesophageal varices,arteriovenous malformations, vascular aneurysms or major intraspinal or intracerebral vascular abnormalities.	active clinically significant bleeding	unclear	not codable
	lesion, if considered to be a significant risk for major bleeding	unclear	not codable
	condition, if considered to be a significant risk for major bleeding	unclear	not codable
	current gastrointestinal ulceration	clear	advanced
	recent gastrointestinal ulceration	unclear	not codable
	presence of malignant neoplasms at high risk of bleeding	unclear	not codable
	recent brain injury	unclear	not codable
	recent spinal injury	unclear	not codable
	recent brain surgery	unclear	not codable
	recent spinal surgery	unclear	not codable
	recent ophthalmic surgery	unclear	not codable
	recent intracranial haemorrhage	unclear	not codable
	known oesophageal varices	clear	basic
	suspected oesophageal varices	clear	not codable
	arteriovenous malformations	unclear	advanced
	vascular aneurysms	unclear	advanced
	major intraspinal vascular abnormalities	unclear	not codable
	major intracerebral vascular abnormalities	unclear	not codable

SmPC (original text)	Individual absolute CIs (extraction)	Clarity	Codability
Concomitant treatment with any other anticoagulants, e.g. unfractionated heparin (UFH), low molecular weight heparins(enoxaparin, dalteparin, etc.), heparin derivatives (fondaparinux, etc.), oral anticoagulants (warfarin, dabigatran etexilate,apixaban, etc.) except under specific circumstances of switching anticoagulant therapy (see section 4.2) or when UFH is given at doses necessary to maintain an open central venous or arterial catheter (see section 4.5).	other anticoagulants (concomitant treatment), except under specific circumstances of switching anticoagulant therapy	clear	not codable
	unfractionated heparin (UFH) (concomitant treatment), except under specific circumstances of switching anticoagulant therapy or when UFH is given at doses necessary to maintain an open central venous or arterial catheter	clear	not codable
	low molecular weight heparins (concomitant treatment), except under specific circumstances of switching anticoagulant therapy	clear	not codable
	enoxaparin (concomitant treatment), except under specific circumstances of switching anticoagulant therapy	clear	not codable
	dalteparin (concomitant treatment), except under specific circumstances of switching anticoagulant therapy	clear	not codable
	heparin derivatives (concomitant treatment), except under specific circumstances of switching anticoagulant therapy or when UFH is given at doses necessary to maintain an open central venous or arterial catheter	clear	not codable
	fondaparinux (concomitant treatment), except under specific circumstances of switching anticoagulant therapy	clear	not codable
	oral anticoagulants (concomitant treatment), except under specific circumstances of switching anticoagulant therapy	clear	not codable
	warfarin (concomitant treatment), except under specific circumstances of switching anticoagulant therapy	clear	not codable
	dabigatran etexilate (concomitant treatment), except under specific circumstances of switching anticoagulant therapy	clear	not codable
	apixaban (concomitant treatment), except under specific circumstances of switching anticoagulant therapy	clear	not codable
Hepatic disease associated with coagulopathy and clinically relevant bleeding risk including cirrhotic patients with Child Pugh B and C (see section 5.2).	hepatic disease associated with coagulopathy and clinically relevant bleeding risk	unclear	not codable
	cirrhotic patients with Child Pugh B	clear	basic
	cirrhotic patients with Child Pugh C	clear	basic
Pregnancy and breast-feeding (see section 4.6).	pregnancy	clear	not codable
	breast-feeding	clear	not codable

CI: contraindication, SmPC: Summary of Product Characteristics
